# Estimating species commonness and prevalence through unsupervised methods

**DOI:** 10.1038/s41598-026-38900-1

**Published:** 2026-02-11

**Authors:** Pasquale Bove, Andrea Bertini, Gianpaolo Coro

**Affiliations:** 1 Istituto di Geoscienze e Georisorse, Consiglio Nazionale delle Ricerche, 56124 Pisa, Italy; 2 Independent Researcher, 56126 Pisa, Italy; 3 Istituto di Scienza e Tecnologie dell’Informazione “A. Faedo”, Consiglio Nazionale delle Ricerche, 56124 Pisa, Italy

**Keywords:** Species commonness, Species prevalence, Artificial intelligence, Deep learning, Wetlands, Computational biology and bioinformatics, Ecology, Ecology, Environmental sciences

## Abstract

The prevalence of a species in a given area is crucial for estimating the environmental conditions associated with its subsistence within ecological niche models (ENMs). Prevalence is defined as the proportion of presences relative to the total number of sampled sites, reflecting prior expectation on species commonness or rarity. However, reliable estimation often faces challenges due to limited or biased occurrence data, particularly for rare or poorly monitored species. This work presents a data-driven, multi-species methodology to estimate species prevalence for use in ENMs. It leverages species occurrence records from the Global Biodiversity Information Facility and is entirely unsupervised. It utilises two clustering methods, one deep-learning model, and an ensemble model, plus statistical analysis to classify species commonness and transform classifications into prevalence probabilities. A case study is presented for 161 species living in the Massaciuccoli Lake basin (Tuscany, Italy), a wetland of high biodiversity value and ecological sensitivity. The models classified the species’ prevalence based on observations from other Italian wetland sites, and were evaluated against expert-based assessments. All models achieved high accuracy, with the deep-learning model achieving the highest (~ 81–90%). The proposed methodology is scalable and reproducible and can inform ENMs with objective, robust prevalence estimates.

## Introduction

Ecological Niche Models (ENMs) estimate the array of resources and environmental conditions that facilitate the persistence and proliferation of a species within a specific area, commonly referred to as the species’ *ecological niche*^1–7^. These models can operationally define a species’ suitable habitat as the locations within the study area that possess suitable environmental conditions corresponding to the species’ *ecological niche*. Consequently, ENMs are also capable of predicting the potential presence or absence of a species in an area different from its native area (the *potential ecological niche*)^8,9^. From a mathematical perspective, a species’ ecological niche can be conceptualised as a region in a vector space (a *hypervolume*) formed by the environmental variables relevant to the species’ survival.

The efficacy of ENMs is contingent upon the accurate identification of a comprehensive set of environmental variables that constitute the dimensions of this vector space, as well as the availability of prior information regarding the species’ presence in the area^1,10,11^. This prior information (*species prevalence*) is particularly crucial for driving an ENM towards the correct estimation of the ecological niche, as it defines the boundary conditions and starting assumptions for the model’s fitting to the data. Generally, species prevalence is pivotal in both the calibration and interpretation of ENMs^12,13^. Formally, it is defined as the proportion of presence data relative to the total number of sites examined while surveying, considering both presences and absences (or pseudo-absences)^14,15^. Therefore, it also indicates the extent of the landscape that is occupied or deemed suitable for the species under consideration, before fitting an ENM to the species observation data.

The relevance of species prevalence for ENMs is evident in two primary aspects. First, it directly affects the calibration of model outputs, particularly when algorithms generate continuous suitability scores that can be transformed into probabilities of occurrence^15–18^. For instance, in Bayesian models, such as Maximum Entropy (MaxEnt)^19–21^, assumptions regarding species prevalence influence the conversion of raw suitability scores into logistic probabilities of species presence. Within these frameworks, prevalence acts as a parameter that introduces prior information into the models concerning the anticipated species presence in the focus area. Species prevalence also corresponds to the prior probability of the species’ presence in the area, indicating the expectation of whether the species is rare or common in that area prior to observation^22–24^. Variations in prevalence settings could therefore alter the modelled species habitat distributions.

Second, species prevalence has a considerable impact on the process of thresholding continuous predictions into binary presence-absence maps^10,25,26^, where the selection of optimal thresholds often depends on the equilibrium between sensitivity (true positive fraction) and specificity (true negative fraction). This balance is inherently influenced by species prevalence. Many metrics used for evaluating ENM performance (e.g., Kappa, True Skill Statistic) indeed display increased sensitivity to imbalances between presences and absences^25,27–29^. This sensitivity is particularly relevant for rare species characterised by low prevalence, as data scarcity may hinder predictive performance and obscure niche boundaries. Prevalence indeed embodies ecological characteristics indicative of species rarity or commonness^30–33^. Consequently, a careful consideration of prevalence is paramount for generating robust, interpretable, and ecologically meaningful niche models.

**Fig. 1:**
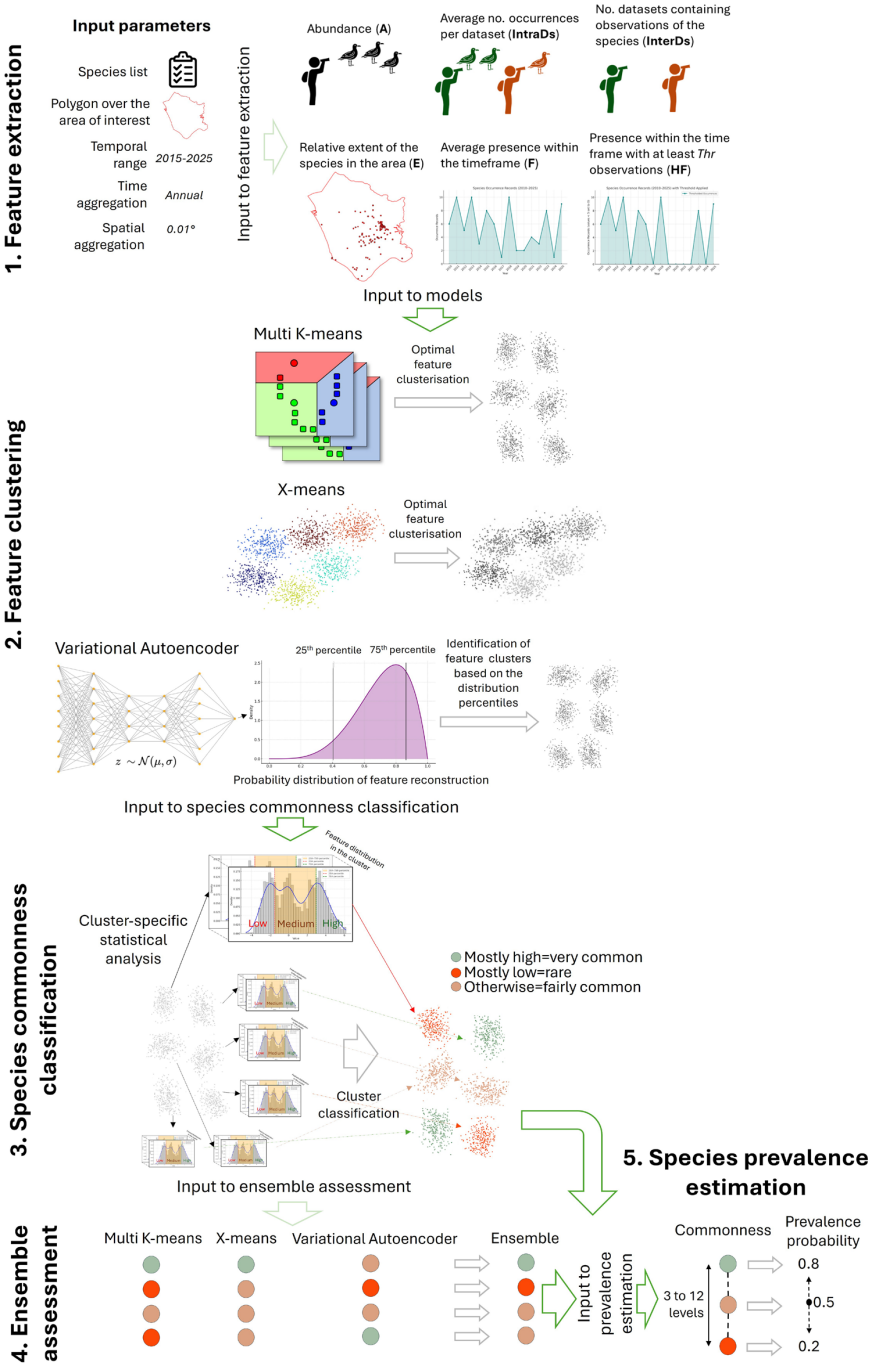
Overview of the methodological workflow.

Given the importance of prevalence for ENMs, it is paramount that its estimate is as objective as possible, unaffected by individual conjectures that might be subject to biases. This paper presents a data-driven, multi-species methodology for objectively estimating species prevalence (Fig. 1). The methodology builds upon species occurrence records from the publicly accessible Global Biodiversity Information Facility (GBIF^34^), while addressing issues related to the possible under-representation of species presence in the analysed area. It uses three different unsupervised machine learning techniques, comprising two cluster analysis methods (Multi K-means and X-means) and one deep learning model (a Variational Autoencoder, VAE). These models process aggregative features for each species under analysis, defined on the species occurrence records. These features highlight the spatio-temporal coverage of the occurrences, the frequency of observation, and the number of diverse data collections that reported presence in the area under analysis. The model outputs are statistically analysed to classify the commonness level of each species, which is subsequently transformed into species prevalence probability. The cluster analysis methods yield discretised values corresponding to classes of species commonness, while the deep learning model provides a continuous score that is eventually discretised for classification.

**Fig. 2:**
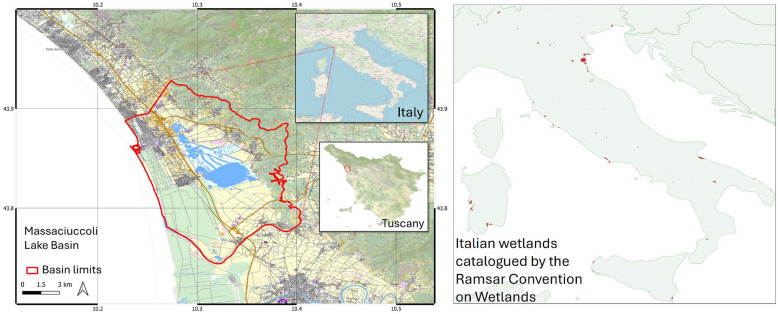
Overview of the Massaciuccoli Lake basin (left image) and the Italian wetlands from the Ramsar Convention on Wetlands (red areas in the right image). Maps were created with QGIS v.3.40 (URL: www.qgis.org). The Italy map was the OSM standard map of the QuickMapService plugin. The Tuscany map was taken from the Tuscany Regional Government online GIS service (URL: www502.regione.toscana.it/geoscopio/cartoteca.html). The Ramsar wetland areas were taken from the Repertorio Nazionale dei Dati Territoriali (URL: geodati.gov.it/resource/id/m_amte:299FN3:99622692-1e7a-452b-86d7-3ae4fe96c385).

To evaluate and illustrate the effectiveness of the methodology, a case study centred on the Massaciuccoli Lake basin in Tuscany, Italy, is presented (Fig. 2). This basin is a wetland and a significant tourist attraction, distinguished by its rich biodiversity. The hydrogeological, chemical, and environmental characteristics of the basin are critical in determining the habitat suitability for the presence and persistence of both native species (e.g., black bullhead, eel, tench, largemouth bass, carp, heron, and kingfisher) and non-native species (e.g., sheatfish and red swamp crayfish). Additionally, the basin functions as a crucial stopover point for numerous bird migrations, thus garnering the interest of citizens, tourists, and scientists^35,36^. Here, as generally in wetland ecosystems, birds are particularly vulnerable to changes in biogeochemical and hydrological conditions within the current climate change dynamics^37–40^. Species prevalence plays a pivotal role in predicting biodiversity patterns within this region over short and long time scales.

The three models for species commonness classification were applied to numeric features calculated from the occurrence records of 161 species of the Massaciuccoli Lake basin over the past decade (2015-2025). Model training utilised GBIF data from the Italian wetlands catalogued by the Ramsar Convention on Wetlands^41^ (Fig. 2-right panel). Alternative species-commonness assessments were conducted by two local experts and used as references for evaluating model classification performance. Additionally, a sensitivity analysis of the models on the features was conducted using a leave-one-out procedure to highlight those with the highest importance for classification.

### Overview

The term *common species* denotes a species that exhibits high abundance in a specific area, has a widespread distribution, and is considered at low risk of extinction^42–44^. In contrast, *rare species* are characterised by lower abundance, unsuitable habitat, or potential threats to their survival. Automatic detection and monitoring of common and rare species is crucial for understanding the implications of environmental change on ecosystem functioning. A species’ abundance serves as an indicator of its ecological role and is influenced by the relative abundances of other species^45–47^. Rare species often contribute significantly to biodiversity due to their unique functional traits^48–52^, while common species underpin ecosystem function through their biomass dominance^42,53–59^.

In ENMs, species commonness can be used to estimate species prevalence^16,18,60,61^. Traditional ENMs, like MaxEnt, require prevalence to transform relative occurrence rates into posterior probabilities of presence^21,62^. Bayesian approaches also utilise prevalence as an uncertain parameter, enhancing the reliability of species range predictions^63–65^. These analyses also highlight the impacts of environmental changes on relative species abundances^66–68^.

Monitoring biodiversity changes requires large-scale, multi-species ecological analyses^69–72^. Meta-analyses across studies can assess the commonness and prevalence of multiple species within specified timeframes^73–75^. Researchers can leverage extensive biodiversity databases, such as the United Kingdom’s National Biodiversity Network^76^, the Global Biodiversity Information Facility, or the Ocean Biogeographic Information System (OBIS)^77^, which provide millions of records detailing species distributions over time. However, extracting robust estimates from these heterogeneous data sources poses significant challenges^78–81^.

A primary concern is the difficulty of distinguishing the actual prevalence of a species from the noise introduced by varying sampling efforts that are temporally, spatially, and taxonomically focused^82^. A species may appear abundant during a specific decade in a large dataset due to intensive sampling efforts focused on that species. A subsequent decline in apparent abundance may simply reflect the end of that programme, lacking ecological significance. The presence of a species’ occurrence records in large collections is influenced by various factors, including (i) sampling frequency, (ii) ease of observation, (iii) whether the species is targeted in surveys, and (iv) the temporal extent of those surveys. Such biases may significantly impact assessments made by models or experts regarding species prevalence. Consequently, the information in large datasets generally represents a partial reality.

To mitigate these challenges, several methodologies integrate species observation records with supplementary information from expert documents or other databases^83–88^. While these approaches address biases, they may only be generalizable to species for which supplementary data exist, potentially excluding underrepresented species. Some approaches have introduced analytical definitions of species commonness based on subjective expert knowledge^22,89^, yet a universally satisfactory definition remains elusive. Therefore, assessing species commonness often necessitates the identification of limited commonness classes, and since species prevalence relates closely to commonness, prevalence probability is also discretised. This does not necessarily diminish the accuracy of information conveyed to multi-species ENMs. For instance, cluster analysis has proven effective for detecting multi-species presence patterns^33,90–94^. Furthermore, accuracy in prevalence discretisation can be enhanced through re-analysis of presence-only ENMs, expert assessments, or independent surveys^15,95–97^. Discretised prevalence ranges are also useful for estimating statistical uncertainty in habitat suitability probabilities estimated by ENMs^27,98–100^.

Despite the importance of species prevalence for ecological niche modelling, current approaches face limitations when applied to large, diverse species sets. Many methods rely on species-specific modelling, external ecological traits, or expert-defined thresholds to address incomplete occurrence data, leading to subjectivity and limiting scalability. Additionally, prevalence is often discretised into fixed categories without a consistent rationale. These challenges highlight the need for a framework that can jointly assess prevalence across multiple species, operate under incomplete data, and minimise expert reliance.

The unsupervised methodology proposed in this paper addresses this gap by utilising aggregated, ecologically meaningful features from occurrence data, enabling direct comparison across species without assuming completeness. Clustering and anomaly-detection techniques serve to identify dominant prevalence patterns and detect deviations in rare or undersampled species. This design enables prevalence to emerge from the data structure while preserving flexibility in discretisation and ensuring reproducibility across taxa.

In summary, the methodology presented in this paper incorporates effective strategies suggested by other works to offer a novel approach that incorporates the following features: (i) it generates objective multi-species prevalence assessments; (ii) accounts for the knowledge gaps inherent in extensive collections of species occurrence records; and (iii) employs prevalence discretisation with adjustable granularity.

## Results

### Classification performance

The VAE demonstrated the highest level of concordance with all expert assessments, both in the binary and three-category evaluations (Table 1). It achieved the greatest accuracy when compared to the expert ensemble, recording values of 90.06% (146 species assessed correctly) and 85.71% (138 species assessed correctly) in the two evaluations, respectively. Notably, a *substantial* agreement (following Fleiss’ interpretation of Kappa^101^) was measured in these cases, with Kappa of 0.80 and 0.76, respectively. The VAE classified 71 species as very common (69 correctly), 51 as fairly common (51 correctly), and 39 as rare (18 correctly). A higher agreement with the “expert ensemble” was observed across all models, with an average relative increase of 10% accuracy compared to the lowest agreement. The minimum level of agreement was consistently associated with Expert 1, with whom the VAE reached a moderate/fair agreement, achieving accuracies of 81.37% and 70.81% and Kappa of 0.63 and 0.54 in the two evaluations, respectively. Expert 1 generally tended to assign higher commonness levels than the models. The agreement with Expert 2 was intermediate between the others, with the VAE achieving accuracies of 89.44% and 85.09% in the two evaluations and a substantial agreement (Kappa = 0.79 and 0.75, respectively).

The high concordance with the “expert ensemble” can be attributed to the ensemble methodology; when there was significant disagreement between the experts regarding a species commonness classification, the ensemble opted for a moderate classification (“fairly common”). This approach effectively harmonised the differing perspectives of the experts. Overall, expert disagreement occurred for only a lesser fraction of the species (20%). The uncertain cases mainly involved species whose prevalence and observational frequency were on the cusp of being classified as either very common or fairly common. The results revealed a tendency for all models to categorise species as “fairly common” in cases where the experts expressed uncertainty. However, the predominant classification overall was that of very common species.

The fact that the VAE achieved the highest overall accuracy underscores the correlation between *anomalies* in the feature values and species *rarity*, thereby validating the hypothesis explained in “Methods” (section “Classification”): the characterisation of rare species is more varied than that of common species.

Table 1 also indicates that Multi K-means (with $K^*$=5) yielded a lower average accuracy compared to X-means (with $K^*$=7) in both binary (83.6% vs. 84.5%, on average) and three-category (77.43% vs. 79.30%, on average) classifications. Agreement with the experts ranged from moderate to substantial. These findings suggest that the slightly higher fragmentation of the feature space by the X-means model, as determined by the BIC, was more effective in highlighting species-commonness characteristics within each cluster. Generally, the cluster analysis models achieved high agreement with the experts, although they were less accurate than the VAE.

The ensemble model achieved an average accuracy of 84.5% in binary classification and of 75.8% in three-category classification, exhibiting moderate to substantial agreement with the experts. This model was predominantly influenced by the cluster analysis models. The VAE provided particular support in binary classification, shifting decisions towards the X-means assessments in cases of disagreement between the cluster analysis models. As a result, the ensemble assessment generally resembled an enhanced variant of Multi K-means.

As an additional validation, prevalence scores derived from X-Means were utilised within a multi-species ENM to assess biodiversity trends in the Massaciuccoli basin (described in^60^). This model exhibited considerable robustness, with respect to evidence, in delineating the current species richness scenario (2016–2024) compared to both a remote past (1950–1980) and a more recent temporal context (1981–2015). Furthermore, it successfully projected biodiversity scenarios for both short-term (2025–2050) and long-term (2051–2100) future perspectives. These findings underscored the effectiveness of reliable prevalence discretisation, particularly in the context of big data-driven multi-species modelling.

**Table 1:** Performance comparison (accuracy and Cohen’s Kappa) in species commonness classification between the proposed models, two experts, and an ensemble expert assessment. The comparisons are reported for binary classification (very common vs. less common species) and three-category classification (very common, fairly common, or rare).

	Two categories							
	Multi K-means		X-means		VAE		Ensemble	
	Acc. (%)	Kappa	Acc. (%)	Kappa	Acc. (%)	Kappa	Acc. (%)	Kappa
Expert 1	78.26	0.57	77.64	0.55	81.37	0.63	78.88	0.59
Expert 2	85.71	0.71	87.58	0.75	89.44	0.79	86.96	0.74
Expert ensemble	86.96	0.73	88.20	0.76	90.06	0.80	87.58	0.75

	Three categories							
	Multi K-means		X-means		VAE		Ensemble	
	Acc. (%)	Kappa	Acc. (%)	Kappa	Acc. (%)	Kappa	Acc. (%)	Kappa
Expert 1	68.32	0.52	69.57	0.50	70.81	0.54	65.22	0.47
Expert 2	81.37	0.70	83.85	0.73	85.09	0.75	80.75	0.68
Expert ensemble	82.61	0.72	84.47	0.73	85.71	0.76	81.37	0.69

### Sensitivity analysis

The leave-one-out analysis explained in “Methods” (section “Evaluation methodology”) demonstrated that each feature significantly contributed to both accuracy and agreement (Table 2). The removal of any single feature invariably led to a reduction in both metrics. The Kappa reductions provided a clearer delineation of the variable rankings, from the feature with the most substantial information content to that with the least influence. The accuracy losses ranged from -5.56% to -3.17% for binary classification and from -8.18% to -3.64% for the three-category classification. In both classification scenarios, the most important variable was the average number of occurrence records per dataset (*IntraDs*). Species frequently documented across multiple wetlands were likely to be classified as “very” or “fairly” common. Conversely, the mere fraction of datasets containing species observations (*InterDs*) was less critical compared to the other features, despite its positive contribution to performance.

The average species abundance per occurrence record (*A*) was another important parameter, since very abundant species are often common species. The extent of species presence in the areas (*E*) was another important feature for binary classification, although it was less important for three-category classification. A species occupying most of the area was likely to be associated with a “very common” species, but was less crucial to distinguish between “fairly common” and “rare” species.

The importance of observation frequencies (*F* and *HF*) was different when transitioning from binary to three-category classification. In binary classifications, the roles of these variables were marginal. In contrast, they became pivotal in distinguishing between “fairly common” and “rare” species within the three-category classification. In particular, the thresholded observation frequency (*HF*) exhibited a substantial shift in importance ranking, rising from the 6th to the 2nd position. The contribution of *F* to accuracy was also greater in the three-category classification (-5.45% decrease) than in the binary classification (-3.97% decrease). Overall, maintaining a consistently high frequency of observations over the years proved to be crucial in accurately classifying “fairly common” species.

**Table 2:** Average percentage reduction of accuracy and Cohen’s Kappa for the ensemble model when excluding one feature at a time (leave-one-out process). The expert ensemble assessments were used as the references.

	Relative decrease	
Excluded feature	Accuracy (%)	Kappa
Two categories		
IntraDs	− 5.56%	− 17.10%
A	− 5.56%	− 16.23%
E	− 3.97%	− 13.61%
F	− 3.97%	− 13.14%
InterDs	− 3.97%	− 11.88%
HF	− 3.17%	− 10.53%

Three categories		
IntraDs	− 8.18%	− 14.57%
HF	− 8.18%	− 14.02%
A	− 7.27%	− 13.91%
F	− 5.45%	− 8.99%
E	− 4.55%	− 8.97%
InterDs	− 3.64%	− 8.41%

### Analysis of the misclassified species

**Fig. 3:**
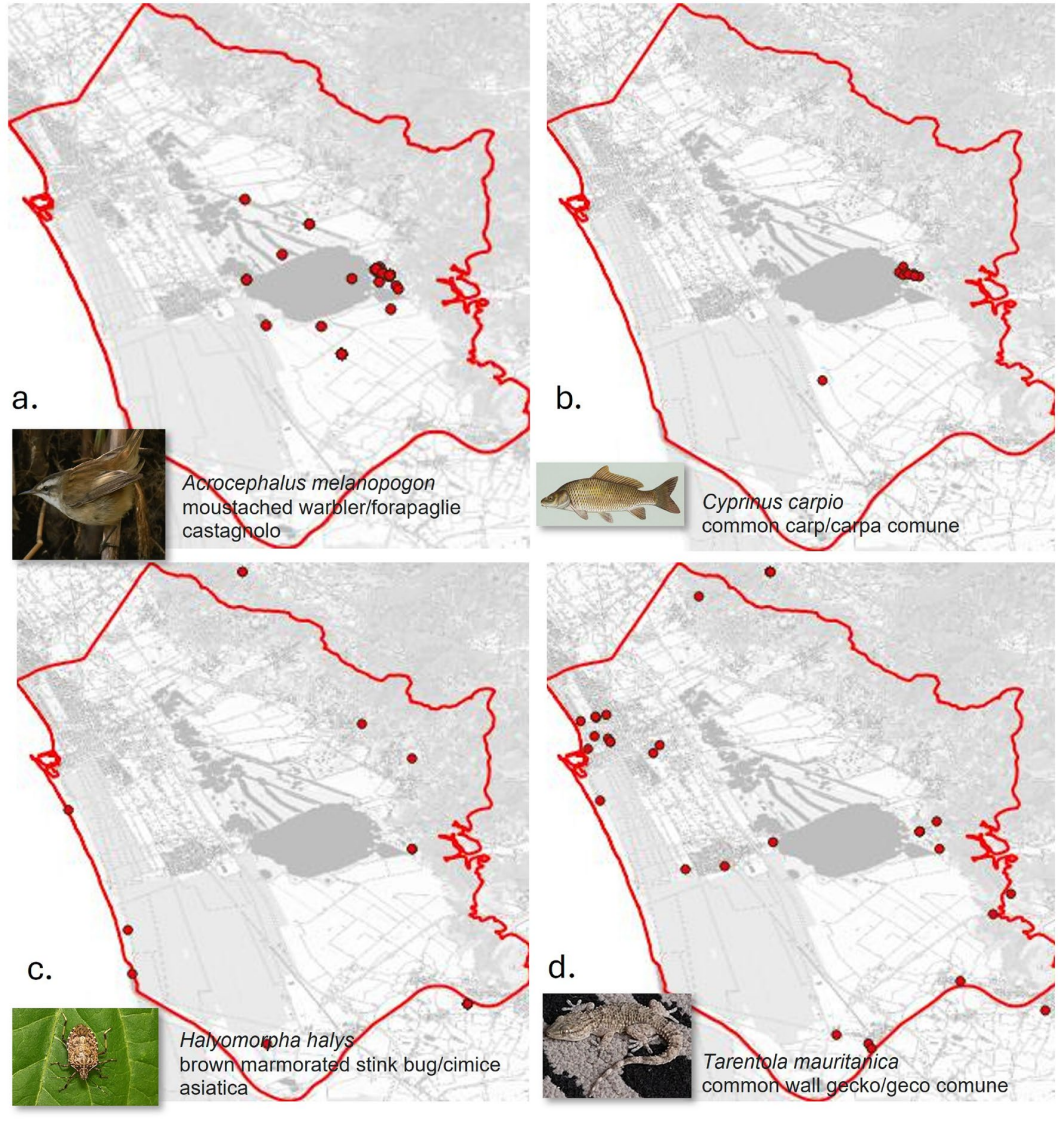
Species for which maximum disagreement between the experts and the models was observed: the experts indicated the species as very common, whereas the models indicated rarity. The red dots illustrate the spatial distributions of the species occurrence records in GBIF (URL: www.gbif.org). Maps were created with QGIS v.3.40 (URL: www.qgis.org). The Tuscany background map was taken from the Tuscany Regional Government online GIS service (URL: www502.regione.toscana.it/geoscopio/cartoteca.html - Carta Topografica). The species’ images were taken from Wikipedia (URL: co.wikipedia.org/wiki/Acrocephalus_melanopogon#/media/File:Moustached_Warbler_(Acrocephalus_melanopogon)_(33906770030).jpg, URL: it.wikipedia.org/wiki/Cyprinus_carpio#/media/File:Common_carp.jpg, URL: it.wikipedia.org/wiki/File:Halyomorpha_halys_lab.jpg, and URL: it.wikipedia.org/wiki/Tarentola_mauritanica#/media/File:Gekon_murowy_(Tarentola_mauritanica).jpg).

Four species exhibited a consistent divergence between expert assessments and model predictions, with the experts evaluating them as “very common” species and the models indicating them as “rare” species (Fig. 3):

1. One species was *Acrocephalus melanopogon* (Temminck, 1823), the moustached warbler (*forapaglie castagnolo*, in Italian) (Fig. 3a). This bird primarily breeds in southern Europe and southern-temperate Asia. GBIF has collected 82 records of this species over the past decade within the Massaciuccoli basin, with a significant concentration of records in the lake area. Additionally, approximately 40 records were available from the other Italian wetlands. Although *A. melanopogon* is considered elusive within the Massaciuccoli basin, it encounters a particularly suitable habitat there, more than in other wetlands^102^. Observations documented in GBIF were predominantly concentrated in the months of April and May. The observations suggested that the species was abundant around the lake, but the average frequency of observations was relatively low (only five observations per year). The experts classified the species as “very common” due to its well-established presence in the area, whereas the models deduced rarity based on year-round presence and data from other wetlands. This case highlights that data derived from other wetlands do not always provide reliable insights regarding the specific wetland under analysis.
2. Another species was *Cyprinus carpio* (Linnaeus, 1758), the common carp (*carpa comune*, in Italian) (Fig. 3b). Across Europe, this fish is prevalent in eutrophic freshwater habitats, including lakes and large rivers^103^. Often classified as invasive and detrimental in regions where it has been introduced, *C. carpio* presents a contrasting scenario in the Massaciuccoli basin, where it is not perceived as a problematic species. Its presence is well-documented, and the population appears to be stable^104^. GBIF has recorded merely 12 observations over the last decade, and a similar number across the other Italian wetlands. Thus, the experts’ assessments were influenced by the general classification of the species as invasive, while the models relied on the data.
3. The third species was *Halyomorpha halys* (Stål, 1855), the brown marmorated stink bug (*cimice asiatica*, in Italian) (Fig. 3c). This insect is indigenous to various regions in Asia, particularly China, Japan, and Korea, and is recognised for its highly invasive nature in non-native habitats, where it poses a threat to agricultural crops^105^. For the Massaciuccoli basin, GBIF contained 19 observations from the last decade, primarily concentrated along the shoreline and in rural towns. Although the presence of this species in the basin has been documented through scientific surveys and is believed to result from accidental introductions^106^, there is currently no indication of an established invasion. Comparable numbers of observations and distribution patterns were available across the other Italian wetlands as well. Consequently, in this instance, the expert assessments were predominantly influenced by the known invasive behaviour of the species.
4. The fourth species was *Tarentola mauritanica* (Linnaeus, 1758), the common wall gecko (*geco comune*, in Italian) (Fig. 3d). This nocturnal reptile is frequently observed inhabiting walls within urban settings in temperate coastal regions. In Italy, similarly to other southern European nations, it has developed a longstanding association with humans, primarily as a predator of insects^107^. GBIF contained a total of 40 observations for the Massaciuccoli basin, which were notably sparse and predominantly located in urbanised areas. An average of 15 observations was available for each of the other Italian wetlands. The frequency of observations on a yearly basis remained low, with a significant concentration of reports in the past four years. For this species, the assessments conducted by the experts were based on the species’ widespread distribution in Tuscany and throughout Italy, which was not reflected in the data.

A lower level of divergence in species commonness classification between the models and the experts was noted for a few other species. These cases encompassed borderline species positioned at the threshold between two classification categories. One group included species regarded by experts as “very common” but classified as “fairly common” by the models, which were *Acrocephalus arundinaceus* (the great reed warbler/*cannareccione*) and *Acrocephalus schoenobaenus* (the sedge warbler/*forapaglie*). Another group included borderline-abundant or elusive species that the experts classified as “rare”, whereas the models categorised as “fairly common”, which were *Ichthyaetus melanocephalus* (the Mediterranean gull/*gabbiano corallino*), *Haematopus ostralegus* (the Eurasian oystercatcher/*beccaccia di mare*), and *Plegadis falcinellus* (the glossy ibis/*mignattaio*). In these instances, the significant subjectivity inherent in the assessment process was the principal reason for the observed discrepancies.

## Discussion

This study has presented a novel, data-driven, multi-species methodology for classifying species commonness and subsequently estimating species prevalence to serve as prior probability in ENMs, particularly in contexts where the only data available are species occurrence records from large collections. The methodology utilises occurrence records from GBIF, regarding ecologically similar regions or, alternatively, multiple surveys and data collections. It employs unsupervised methods for processing these data, including cluster analysis (Multi K-means, X-means) and deep learning (Variational Autoencoders, VAE). Moreover, it uses statistical analysis to classify species commonness and translate these classifications into prevalence probabilities. Being completely data-driven, the methodology ensures that the assessments of species commonness and prevalence are not subjective. Consequently, the presented approach ensures the provision of objective and neutral prior prevalence information over multiple species for ENM and biodiversity assessments, thereby contributing to the reproducibility and transparency of habitat distribution estimates. The methodology is designed for direct reusability, enabling quick prior estimates for numerous species. The utilisation of the publicly accessible GBIF data, coupled with standardised feature extraction, ensures that this methodology can be applied across a wide array of ecosystems and taxa, thereby enhancing its scalability and reusability, and potentially achieving global coverage.

Validation of the methodology was presented via a case study conducted in the biodiversity-rich wetland of the Massaciuccoli Lake basin (Tuscany, Italy). The models were trained on GBIF data sourced from other Italian wetland sites and subsequently evaluated against expert-based assessments of species commonness. The VAE demonstrated superior accuracy, achieving up to 90.06% in binary classification (“very common” vs. “less common”) and 85.71% in three-category classification (‘very common”, “fairly common”, or “rare”), along with substantial agreement with expert consensus. This confirmed the model’s effectiveness in capturing patterns of species rarity and commonness. Notably, the correspondence between anomalous features and species rarity suggested a more varied characterisation of rare species compared to common species.

The results highlighted the Multi K-means model’s tendency to assess lower commonness levels, as the ($UNIF(K)$) optimisation function, internally used by this model, fostered the formation of large clusters that included a wider range of feature values. The ensemble model exhibited a similar tendency but did not yield a statistically significant improvement in performance. On the contrary, X-means fragmented the feature space into slightly smaller clusters, making the classification more reliable. It also showed a tendency towards assessing higher commonness levels. The VAE yielded higher classification performance, but an important requirement emerged for its proper application to other scenarios: the species list must be sufficiently extended and varied so that anomalies can enable the detection of rare species. Consequently, X-means would be preferable in data-poor and limited-species scenarios. Finally, the sensitivity analysis underscored the relative importance of various features, identifying intra-dataset record abundance, species abundance per record, and observation frequency as key indicators of species commonness.

Although the analysis was demonstrated on this case study, the proposed framework is designed to be transferable to other ecological contexts, provided that comparable occurrence-derived features can be computed and that the species set is sufficiently large and heterogeneous. Generality is thus claimed at the level of the methodological approach rather than at the level of numerical results or ecological thresholds, which are expected to vary across systems.

One critical aspect of our methodology concerns the spatio-temporal aggregation of the observations, which can impact classification outcomes. Aggregating data on an annual basis results in the loss of differentiation between permanent and migratory species. Furthermore, employing a decadal time frame for occurrence retrieval penalises the classification of short-lived species. Additionally, conducting aggregations at a broad spatial resolution restricts the ability to identify species that may be prevalent at sub-area scales. Therefore, optimising spatio-temporal aggregation is critical for enhancing the granularity of assessments and facilitating more thorough investigations into specific species.

An intrinsic limitation of the proposed methodology is the unavoidable presence of spatial, temporal, and taxonomic sampling biases. Although the implementation of aggregative features, in conjunction with multi-clustering and anomaly analyses, alleviates this issue, these biases still influence prevalence estimates, particularly for elusive or underreported species. Additional misclassification can occur for species with borderline commonness status. The reliance on potentially biased information necessitated a compromise on the accuracy of the classifications and prevalence estimations. However, the methodology allows for increasing the resolution of the classes (up to 12), thereby enabling the exploration of sub-classes and more accurate prevalence values. Nevertheless, the basic three commonness classes used in the case study have already exhibited considerable informativeness for ENMs in data-limited scenarios, as evidenced by numerous studies demonstrating their capacity to estimate reliable habitat distributions^3,16,22,60,108–112^. Deeper classification is even less necessary in multi-species models and macroscopic trend analyses^32,113–117^. In data-rich scenarios, instead, the impact of diminished accuracy in prevalence estimation is mitigated by robust model adaptation to the data or by adjusting the estimates through external data.

Future research aimed at refining the methodology will focus on integrating GBIF data with other repositories, citizen science platforms, and field surveys, thereby assessing the necessary efforts to alleviate data biases and enhance feature robustness. Furthermore, the methodology will be expanded to investigate temporal dynamics in species prevalence, supporting ENMs in accounting for ecological changes and anthropogenic impacts over time. Moreover, the impact on commonness classification of a finer temporal granularity in the feature calculation will be investigated for seasonally active species. Lastly, the methodology will undergo testing across diverse ecosystems and taxa to evaluate its generalizability beyond the presented case study and evaluate its applicability in global biodiversity assessments. This testing will be an integral part of our next project proposals.

## Methods

This section outlines our multi-species approach for species commonness classification and prevalence probability estimation (graphically illustrated in Fig. 1). Building upon the lessons learned from different methodologies in species commonness classification^22,33,118–120^, the models presented herein are designed to classify species commonness and subsequently allocate species prevalence probability to each classification. The framework adheres to the strategies for species prevalence discretisation detailed in the “Overview”, which enhance the reliability of prevalence assessments at the cost of reduced granularity.

The methodology principally aims to categorise multiple species into three main commonness classes — *very common*, *fairly common*, or *rare* — based on statistical features derived from species occurrences in the GBIF large data collection. The methodology also permits an increased number of classes, facilitating more nuanced characterisations of species commonness. The basic classes are linked to prevalence probability values of 0.2, 0.5, and 0.8, which serve as multiplicative weights for the likelihood estimated by Bayesian ENMs, informed by species observations and environmental variables. Within this framework, the *medium*-commonness (*fairly common* species) class denotes uninformative prior information. Conversely, the *low*-commonness class (*rare* species) implies that an ENM should adopt a more conservative stance in evaluating species habitat, with a recommendation against assessing locations distant from the observation points. This is reflected in the multiplication of the likelihood by 0.2, effectively lowering habitat suitability assessments in uncertain or low-probability areas. In contrast, the *high*-commonness class (*very common* species) encourages species presence evaluations in areas with fewer observations, introducing a 0.8 multiplicative factor. Notably, setting the high value to 0.8, instead of 1, simulates an intrinsic uncertainty in the assessment (prevalence in the [0.8,1] interval). A more detailed classification is also possible by adding more classes with scores that fall between 0.2 and 0.8 (section “Classification”). This methodology enables quick assessments that yield reliable prior estimates of species prevalence for numerous species.

Our methodology comprises the following principal steps (Fig. 1): feature extraction (section “Feature extraction”), modelling (section “Modelling”), and classification (section “Classification”). During feature extraction, the GBIF observation records pertinent to each species under investigation are sourced. In the modelling step, three models are employed in parallel to analyse the data, organising the features into similarity groups through clustering or anomaly scoring. Finally, in the classification step, a commonness classification is allocated to the groups, thereby distinguishing between variations of species commonness and associating them with respective species prevalence probability values. Given the complementary approaches of the three models, the classification also includes an *ensemble* model that combines the individual-model classifications.

The following sections provide a more detailed explanation of these steps. Moreover, section “Case study” presents the Massaciuccoli Lake basin case study, utilised to assess classification performance across 161 species inhabiting this wetland. Lastly, section “Evaluation methodology” presents the evaluation metrics used for agreement and sensitivity analyses.

### Feature extraction

Among the extensive global-scale data collections of species observations mentioned in the “Overview”, the Global Biodiversity Information Facility (GBIF)^34^ currently encompasses over 3 billion records sourced from more than 116,000 data providers and surveys (commonly referred to as *datasets*) and includes representations of over 8 million species spanning multiple decades. GBIF is widely recognised for its utility in ecological and ecosystem modelling^121,122^, as it satisfies the principles of findability, accessibility, interoperability, and reusability (FAIRness) through the adoption of domain-specific standards, such as the Darwin Core^123^, alongside large-scale e-Infrastructures that offer complementary high-availability access services^124–127^. Furthermore, GBIF incorporates scientific collections and personal archives contributed by researchers, as well as data from citizen science initiatives like iNaturalist^128^ and eBird^129^. Generally, GBIF is an invaluable repository for species observation data. However, it is essential to account for the sampling biases discussed in section “Overview” when inferring species distributions and prevalence from this collection, given that variations in sampling efforts and pathways over time and space may lead to either an overestimation or underestimation of species presence.

Our methodology accesses GBIF through its publicly available R package^130^ to calculate prevalence-related features pertinent to the species under investigation. The definitions of these features are constructed as numerical indicators reflecting the commonness of a species within a defined geographical area and temporal framework. Although a formal definition of species commonness remains elusive, several studies have proposed various features linked to abundance, distribution extent, and the temporal frequency of species observations, which are closely associated with the concepts of rarity and commonness^22,42,131–138^. In these frameworks, a species characterised by high abundance and frequent reporting by multiple data providers (i.e., across various *datasets*) is likely to be commonly encountered within the studied area. The frequency of observation within the specified temporal reference frame serves as an indicator of a species’ persistent presence in the area, potentially correlating with its endemism.

From these studies, it emerges that the input parameters necessary to extract the features required for species commonness characterisation should include (Fig. 1-step 1): (i) a polygon delineating the area of interest, (ii) a temporal framework for the analysis (e.g., from today to 10 years ago), (iii) the temporal granularity of the analysis (*time-unit*), defined as the minimum time interval during which species presence is to be evaluated (e.g., monthly or annually), (iv) a spatial aggregation resolution for the species observations aimed at quantifying species widespreadness in the area occupied, and (v) a list of species for which the features are to be calculated.

Building on previous research regarding the definition of features derived from extensive data collections^22,139^, the formulae in Table 3 were defined. These features originate from possible aggregations of the GBIF data, measuring, for each species, (i) the average number of individuals recorded per observation (*A*), (ii) the total number of observations within the datasets relevant to the study area (*IntraDs*), (iii) the relative number of datasets reporting observations of the species (*InterDs*), (iv) the average widespreadness of the species within the designated area (*E*), (v) the average observation frequency throughout the overall analysis timeframe per specified time-unit (*F*), and (vi) the frequency at which the species appears with “many” observations within a time-unit (*HF*). Notably, these definitions contain parameters that should be adjusted for different geographical regions, i.e., the time frame of the analysis, the time unit, the spatial aggregation for the observations, and the threshold for determining that a number of observations is “relatively high”. Default settings for these parameters can be: a past-decade timeframe, a one-year time unit, a spatial aggregation of $0.01^\circ$ (~1 km), and a threshold of 5 observations for *HF*.

It is important to note that within the definitions, the concept of “dataset” can be effectively substituted with “similar areas”, i.e., regions that exhibit analogous ecosystem characteristics to the area under investigation^140^. For instance, this concept could refer to other wetland regions if the focus is on a specific wetland, or adjacent/similar marine zones in the case of marine ecosystems. Generally, comparable ecosystems may serve as substitutes for datasets in the feature calculations, thereby eliminating the need for diverse, independent surveys conducted for the species in the analysed area.

A notable advantage of the proposed features is that they can be retrieved from various data repositories, in addition to GBIF, such as OBIS^77^ and the Integrated Taxonomic Information System^141^, among others. The only prerequisite is the possibility to access records that include coordinates, the number of individuals, timestamps, and the belonging dataset. Another advantage of using these features is that they are aggregative; therefore, they reduce the bias introduced by individual-record misreporting and wrong localisations through cross-dataset averages and spatio-temporal aggregations. The additional aggregation across multiple species, conducted by the classification (section “Classification”), further contributes to reducing this bias.

In the methodology presented, the occurrence records from GBIF are first pre-processed to remove duplicates (equal coordinates and timestamps). Then, the six features are calculated, for each species under analysis, based on the occurrence records falling within the study area and the input-specified reference time frame (Fig. 1-step 1). These features are then standardised to make their ranges comparable, and compiled into a structured table formatted as a comma-separated-values (CSV) file (*species-data* table). Each row in this table corresponds to one species, while the columns denote the species’ scientific name alongside the values of *A*, *IntraDs*, *InterDs*, *E*, *F*, and *HF*. This table is subsequently utilised as input for each model described in the following section.

**Table 3:** Numeric features extracted from GBIF by our methodology. The term *datasets* refers to the sub-collections available in GBIF.

Feature name	Description	Analytic definition
Species abundance	Average species abundance per occurrence record	$A = \sum\nolimits_{{{\mathrm{datasets}}}} {\frac{{{\mathrm{No}}.\;{\mathrm{individuals}}}}{{No.\;{\mathrm{occurrences}}}}}$
Intra-dataset abundance	Average number of occurrence records per dataset	$IntraDs = \frac{{\sum\nolimits_{{datasets}} {{\mathrm{No}}.\;{\mathrm{occurrences}}} }}{{{\mathrm{No}}.\;{\mathrm{datasets}}}}$
Inter-dataset abundance	Fraction of datasets containing species observations	$InterDs = \frac{{{\mathrm{No}}.\;{\mathrm{datasets}}\;{\mathrm{with}}\;{\mathrm{occurrences}}}}{{{\mathrm{No}}.\;{\mathrm{datasets}}}}$
Extent	Fraction of occurrence cells at the input spatial resolution with occurrence records associated	$E = \frac{{\sum\nolimits_{{{\mathrm{datasets}}\;{\mathrm{with}}\;{\mathrm{occurrences}}}} {\frac{{{\mathrm{No}}{\mathrm{.}}\;{\mathrm{cells}}\;{\mathrm{with}}\;{\mathrm{occurrences}}}}{{No.\;cells}}} }}{{{\mathrm{No}}{\mathrm{.}}\;{\mathrm{datasets}}\;{\mathrm{with}}\;{\mathrm{occurrences}}}}$
Observation Frequency	Average presence in the analysis time frame over the input time-units	$F = \frac{{\sum\nolimits_{{datasets\;with\;occurrences}} {\frac{{No.\;time - units\;with\;occurrences}}{{No.\;time - units}}} }}{{No.\;datasets\;with\;occurrences}}$
High-observation Frequency	Observation frequency with at least input-threshold (*Thr*) observations	$HF = \frac{{\sum\nolimits_{{{\mathrm{datasets}}\;{\mathrm{with}}\;{\mathrm{occurrences}}}} {\frac{{{\mathrm{No}}{\mathrm{.}}\;{\text{time - units}}\;{\mathrm{with}}\;{\text{occurrences > }}Thr}}{{No.\;time - units}}} }}{{No.\;datasets\;with\;occurrences}}$

### Modelling

This section describes the models used to identify aggregational groups for the features extracted in the previous phase. The models reported herein are derived from a selection of clustering and machine learning methodologies that were rigorously experimented in prior ecological-modelling studies^3,22,117,142–144^. Specifically, these models incorporate complementary yet effective approaches that have demonstrated considerable efficacy in species prevalence classification (e.g., Multi K-means and X-means) as well as in ecosystem risk assessment (e.g., Variational Autoencoders). Other methodologies, such as traditional Artificial Neural Networks and alternative clustering techniques, were excluded from consideration due to their lack of complementary features relative to the models selected or insufficient performance reported for species prevalence estimation. Artificial Neural Networks, in particular, were evaluated but did not provide complementary results that enriched the methodology compared to the selected models. The following sections outline the technical specifications of the models selected (illustrated in Fig. 1-step 2).

#### Multi K-means

Cluster analysis is a pivotal data mining methodology aimed at categorising numerical vectors based on their inherent similarities, which are quantified through metrics such as Euclidean distance or density. The Multi K-means method extends the traditional K-means algorithm, enabling clustering across a range of $K$ values (the number of clusters). The K-means algorithm utilises Euclidean distance to allocate data points to clusters. K-means processes the data until convergence is attained. Multi K-means repeatedly applies K-means and eventually estimates the optimal number of clusters, denoted as $K^*$, within a predetermined range. Our methodology uses a $K$ range between 3 and *number of species/2*, to guarantee a minimum distinction between rare, fairly-common, and very-common species, while also exploring a wider array of potential groupings. In each iteration of K-means, initial centroids are randomly chosen from the feature vectors, thereby increasing the probability that the resultant centroids represent realistic conditions.

To evaluate the quality of each clustering and identify the optimal $K^*$, our Multi K-means employs an optimisation function ($UNIF(K)$), which comes from ecosystem risk assessment^117^. This function balances model complexity (i.e., the number of clusters) and the quality of the clustering, checking the uniformity of the distribution of vectors across the clusters. It compares this distribution with a theoretical *uniform* distribution in which the vectors are evenly distributed among the clusters. A Chi-squared test quantitatively assesses the similarity between the population of each cluster and the theoretical distribution. Ultimately, Multi K-means selects the clustering configuration with the best fit, thereby favouring the most homogeneous distribution. Additionally, $UNIF(K)$ incorporates rules to automatically exclude clustering configurations that exhibit empty or sparsely populated clusters (detailed in^117^).

Multi K-means is designed to minimise the risk of over-segmentation and penalise the emergence of sparsely populated clusters. This strategy ensures that the identified clusters are both representative and homogeneous, while preserving the simplicity of the model. A significant outcome of this process is the integration of smaller clusters into larger clusters characterised by similar traits. This merging can potentially yield clusters that are larger than those generated by alternative criteria, such as the Bayesian Information Criterion (BIC)^145^. Different from $UNIF(K)$, the BIC assumes that the optimal clustering is achieved when the vectors are Gaussianly distributed around their respective centroids^146^. In contrast, $UNIF(K)$ does not impose predefined shapes on the clusters.

The output of this model is the association of each species’ feature vector to one cluster among the $K^*$ identified. This association is materialised as cluster indexes reported in a “Multi K-means” column attached to the *species-data* table.

#### X-means

Similarly to Multi K-means, X-means^147^ represents an advancement of K-means that determines the optimal number of clusters through a systematic investigation of a predefined range of $K$. In our methodology, this range is established between 3 and *number of species/2* for consistency with Multi K-means. The algorithm performs K-means clustering for each value of $K$ within this specified range, ultimately selecting $K^*$ as the clusterisation with the minimum BIC value. The minimum BIC balances the goodness of model fit to the data — within the assumption of normal distributions of vectors around the cluster centroids — against the complexity of the model, applying a penalty for excessively complex models to reduce the likelihood of overfitting. X-means was included in our methodology to underscore the impact of utilising BIC as an optimisation criterion as opposed to $UNIF(K)$, because BIC overall tends to favour smaller clusters than $UNIF(K)$.

Similarly to Multi K-means, the model output corresponds to the association of each species’ feature vector with one of the clusters of the optimal clusterisation $K^*$. This association is manifested as an additional “X-means” column in the *species-data* table.

#### Variational autoencoder

A Variational Autoencoder (VAE)^148^ is an unsupervised, deep, probabilistic Artificial Neural Network (ANN) designed to approximate the data distribution $p(\bar{x})$ of a multi-dimensional variable $\bar{x}$. To this aim, it approximates the distribution of latent variables $\bar{z}$ that underlie the generation of the observed data. The architecture of a VAE comprises two principal components: the *Encoder* and the *Decoder*. The *Encoder* is an ANN that maps the input data $\{\bar{x}\}$ to the parameters of a probability distribution over the latent variables $\{\bar{z}\}$. In doing so, it outputs the parameters of a multivariate Gaussian distribution over $\bar{z}$ characterised by mean $\bar{\mu }$ and standard deviation $\bar{\sigma }$. Through this Gaussian distribution, the Encoder approximates the posterior distribution $q(\bar{z}|\bar{x})$. Conversely, the *Decoder* is an ANN with the objective of reconstructing the input data from samples drawn from the latent space ($\bar{z}\sim q(\bar{z}|\bar{x})$). It also simulates the likelihood $p(\bar{x}|\bar{z})$, representing the reconstruction probability of $\bar{x}$ given the latent variable $\bar{z}$. VAEs have found extensive applications in anomaly detection due to their generative capacity and ability to estimate the reconstruction probability of a feature vector^149–151^.

In the methodology presented here, a VAE is utilised as an alternative, independent method to cluster analysis. During model training, the VAE adapts the ANNs to the input feature vectors. This adaptation is accomplished by optimizing a loss function composed of (i) the *Reconstruction Loss*, which assesses the effectiveness of the Decoder in reconstructing the input data (usually measured through mean squared error or cross-entropy); and (ii) the *Kullback-Leibler Divergence*, which encourages the learned posterior distribution $q(\bar{z}|\bar{x})$ to be close to a multivariate Gaussian distribution with $\bar{{\mu }}=(0,\ldots ,0)$ and $\bar{{\sigma }}=(1,\ldots ,1)$.

In our application, the optimal architecture of the VAE is determined automatically during training via the “growing” strategy of ANNs, which aims to identify the configuration that yields the highest overall reconstruction probability across the input vectors, while adding nodes to hidden layers incrementally. The dimensionality of the latent variable $\bar{z}$ is also treated as a tunable hyperparameter and optimised during model training. Specifically, latent dimensions ranging from 1 to the feature dimensionality ($\texttt {dim}(\bar{x})=6$) are systematically evaluated, and the dimensionality yielding the highest reconstruction probability is selected. Notably, searching for a $\bar{z}$ dimensionality lower or equal to that of $\bar{x}$ forces the model to search for compact, disentangled representations of the data that should reduce noise, possibly merge correlated features, and extract dominant generative factors^148^.

The optimal VAE architecture identified for the presented case study (section “Case study”) consisted of 5 neurons in the first hidden layer and 2 neurons in the second. The Decoder mirrored this architecture, comprising 2 and 5 neurons in the two hidden layers, respectively, and a final output layer with 6 neurons. The optimal configuration for $\bar{z}$ corresponded to the latent dimensionality matching the feature dimensionality ($\texttt {dim}(\bar{z})=6$). Lower-dimensional latent spaces resulted in a systematic increase in reconstruction error for both common and rare species, reducing the contrast required for anomaly-based discrimination. This parametrisation of $\bar{z}$ aligned with those found by ecosystem risk assessment models^117^.

A threshold on the reconstruction probability across the training dataset can be employed to distinguish anomalous data (which exhibit lower reconstruction probabilities) from non-anomalous data (which exhibit higher reconstruction probabilities).

In our methodology, the model self-trains on all species-commonness feature vectors and estimates reconstruction probabilities for each vector. The output of the VAE is manifested as a continuous value (reconstruction probability) in an additional “VAE” column within the *species-data* table.

### Classification

To classify species commonness and assign a prevalence score, our methodology employs statistical analysis on the outputs generated by the clustering and VAE models (Fig. 1-step 3).

For the clustering models, the analysis begins by calculating the cluster centroids (mean values per feature in the cluster). Subsequently, it determines the quartiles of the statistical distributions of each feature across the entire dataset. The 25th and 75th percentiles of each distribution are utilized to assign labels to each value of a centroid vector: the label “H” (*high*) is assigned if the value value exceeds the 75th percentile, while the label “L” (*low*) is attributed if the value falls below the 25th percentile; otherwise, the label “M” (*medium*) is assigned. This labelling arises from the understanding that a variable whose average value within a cluster exceeds the 75th percentile (or, equivalently, falls below the 25th percentile) of its distribution across the entire dataset can be considered “high-valued” (or, equivalently, “low-valued”) within that cluster.

The purpose of this labelling is to establish an overall commonness level associated with each cluster. For every cluster centroid, the analysis counts the features categorised as H, M, and L separately. If the H labels dominate, the cluster is categorised as a “high species-commonness” cluster; conversely, if the L labels predominate, it is classified as a “low species-commonness” cluster. All other clusters are designated as corresponding to “medium species-commonness”. The species associated with feature vectors residing within high-commonness clusters are labelled as “very common”, whereas those in the low-commonness clusters are categorised as “rare” and those in the medium-commonness clusters as “fairly common”.

In summary, this classification methodology employs a consensus approach based on the statistical classifications of individual features. A cluster that exhibits high values for the majority of the features is regarded as associated with “high species commonness”, due to the simultaneous occurrence of features with elevated levels. This classification mechanism is also capable of yielding assessments with empty commonness classes; for instance, if no cluster contains a majority of “L” values, the classification will not include “rare” species.

In the final computational step, the classification process assigns a prevalence probability value of 0.2 (rare), 0.5 (fairly common), and 0.8 (very common) to each species’ classification, to be used as a multiplicative weight in Bayesian ENMs.

The consensus mechanism explained also allows for the introduction of more nuanced classifications when the models detect more than three clusters. Specifically, by counting the number of high-valued features (up to a maximum of 6), a 12-level gradient can be created between low and high commonness classifications. This scale encompasses six levels between a full-medium-commonness classification (characterised by all “M” values) and a full-high-commonness classification (comprising entirely “H” values), and other six levels between a full-low-commonness classification (containing all “L” values) and the full-medium-commonness classification. These 12 levels correspond to evenly spaced species prevalence scores ranging from 0.2 to 0.8 (in increments of 0.05). As indicated in the introduction of “Methods”, the necessity for an increased granularity in prevalence assessment should be cautiously evaluated against the sensitivity of the ENMs within the final objectives of the assessment.

A similar classification process is also used for the VAE. Depending on the desired number of species-commonness classes to identify (e.g., 3=low/medium/high commonness), the distribution of the VAE reconstruction-probability across the species is divided into a corresponding number of quartiles. The centroids of the feature vectors residing within each quartile are then calculated, and the same classification procedure described for the clustering models is subsequently executed. This approach stems from the consideration that, provided the species list is sufficiently rich and balanced between rare and common species, anomalous feature vectors are often associated with “rare” species^60^. In quantitative terms, this condition requires (i) a sufficiently large number of species relative to the feature dimensionality, so that the VAE can learn a stable latent representation (as a rule of thumb, at least one order of magnitude more species than features^152,153^), and (ii) a moderate class imbalance, where rare species represent a minority of the dataset (typically below 30–40%). Under these conditions, common species define the dominant structure of the feature space, while rare species populate the lower-density regions of the learned manifold and exhibit lower reconstruction probabilities. The rarity of a species may manifest as particularly low values in one or more features (e.g., low abundance, sparsity, or infrequent observation)^22^, leading to heterogeneous and poorly reconstructed vectors. Consequently, rare species typically form small, dispersed groups that deviate from the reference patterns learned by the VAE. Conversely, when the species list is insufficiently large, highly imbalanced, or lacks feature heterogeneity, the distinction between common and rare species in the reconstruction-probability distribution becomes less pronounced. In such statistically underdetermined conditions, automatic thresholding based solely on quantiles may be unreliable. In these cases, a limited set of expert-identified reference species can be used to calibrate the reconstruction-probability thresholds, assuming that rare species generally exhibit lower reconstruction probabilities than very common species. Importantly, this calibration step does not subvert the data-driven nature of the classification: expert knowledge is used only to anchor the thresholds in scenarios where the available data do not support a stable statistical separation, and the calibrated thresholds are then applied uniformly to all species in the list.

As a concluding step, our methodology creates an *ensemble assessment* that integrates the evaluations provided by the three models (Fig. 1-step 4). This is realised as a consensus model that assigns a commonness classification to each species, corresponding to the classification that receives the majority of assessments from the three models. In instances where no classification achieves a majority, the model defaults to assigning the “fairly common” classification.

In summary, the classification phase assigns four species-commonness labels to each species from the analyses of the Multi K-means, X-means, VAE, and ensemble models, respectively. These labels are then translated into species prevalence estimations (Fig. 1-step 5) and attached to the *species-data* table. This is the final output of the presented methodology.

### Case study

As a case study area, the basin of the Massaciuccoli Lake in Tuscany, Italy, was selected (Fig. 2). This area spans 114 $km^2$, bordered to the North by the Camaiore River, to the East by the Oltre Serchio Mountains, to the South by the Serchio River, and to the West by the Tyrrhenian Sea. The lake itself covers an area of 13 $km^2$ and has a depth ranging from 1.0 to 2.5 *m*, with a total water volume of approximately 14 $Mm^3$^35,154–156^. The primary source of water for the lake is the pluvial regime supplied by the eastern mountains and adjacent agricultural soils. Human activities surrounding the basin have a significant impact on water resources. Agriculture accounts for 40% of these activities, which overall include: farming, cereal and industrial production, horticulture, olive orchards, and railway transportation^157^. Additionally, two wastewater treatment plants operate within the basin, discharging effluents directly into the lake.

The ecological significance of this region is closely linked to its impact on the rich biodiversity, particularly the concentration of bird populations around the lake and its role as a critical hub for migratory routes, which in turn affects related ecosystem services^35,36^. However, the biodiversity of birds residing in or stopping over this area during migration is likely to be adversely affected by climate change^37–40^. Models predict that habitat conditions will become increasingly unsuitable for the majority of bird species by 2100 due to anticipated rises in temperature and aridity^60^. Shifts in the distributions of both terrestrial and aquatic species are expected, driven by alterations in habitat suitability. Overall, climate change presents significant risks to the vulnerability of species inhabiting the Massaciuccoli basin, consequently impacting the ecosystem services and socio-economic values associated with these species^37,40,156,158,159^. In light of these challenges, policy-making authorities are investigating various actions aimed at mitigating these risks^160^, with a principal focus on ensuring the sustained presence of water in the lake. However, these initiatives necessitate robust support from the scientific community to effectively evaluate the timing of ecosystem changes and identify the species most threatened by these alterations. Such assessments can play a critical role in management planning.

To date, scientific investigations concerning the Massaciuccoli Lake basin have primarily focused on statistically analysing the basin’s aggregated species composition and trophic chains^38,39^, alongside specific analyses of vegetation species relevant to the lake’s restoration from eutrophication^37^. Studies on the effects of climate change have examined the long-term impacts of fire and vegetation alterations on current biodiversity^40,161–163^, as well as probing the basin for ongoing geochemical, hydrological, and environmental changes. Notably, one study^60^ has developed future habitat distribution assessments for 180 species utilising ENMs. This research has also underscored the robustness and reliability of such models for aggregated assessments of biodiversity trends, while emphasising the necessity for accurate prevalence assessments to mitigate uncertainty in trend projections and enhance model accuracy.

At the time of this study, GBIF hosts approximately 9,000 records spanning the last decade (2015-2025) for the fauna (Kingdom *Animalia*) residing in the Massaciuccoli basin. These records encompass approximately 400 species from a total of 11 datasets. Among these, 180 species are characterised by non-occasional records and are also present in other Italian wetlands; thus, they can be taken as a reliable reference for animals living in the basin.

**Fig. 4:**
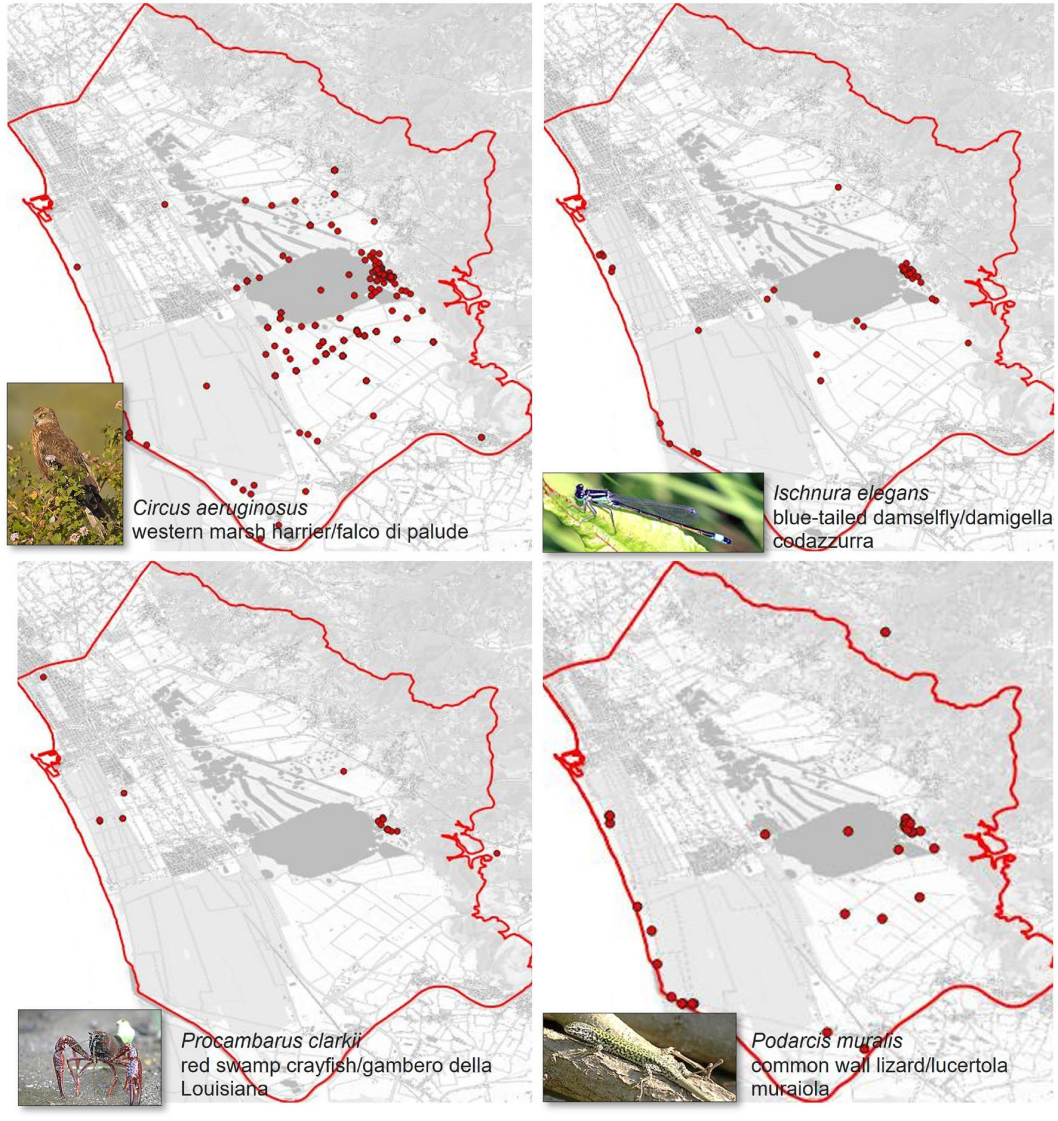
Examples of common species, from different taxonomic groups, living in the Massaciuccoli Lake basin. The red dots illustrate the spatial distributions of the species occurrence records in GBIF (URL: www.gbif.org). Maps were created with QGIS v.3.40 (URL: www.qgis.org). The Tuscany background map was taken from the Tuscany Regional Government online GIS service (URL: www502.regione.toscana.it/geoscopio/cartoteca.html - Carta Topografica). The species’ images were taken from Wikipedia (URL: it.wikipedia.org/wiki/File:Western_Marsh_Harrier-_Bangalore,_India.jpg, cy.wikipedia.org/wiki/Mursen_dinlas_gyffredin#/media/Delwedd:Blue-tailed_damselfly_(Ischnura_elegans)_male_adult.jpg, it.wikipedia.org/wiki/Procambarus_clarkii#/media/File:Procambarus_clarkii.jpg, and it.wikipedia.org/wiki/File:Podarcis_muralis_on_branch.jpg).

Two local experts involved in the present study, who signed informed consent to participate in this research, with decadal experience in identifying the Massaciuccoli species, were able to assess the prevalence of 161 of these species (listed in “Data availability”), thereby enabling an evaluation of our methodology. Of the assessed species, 130 (~ 83%) were birds, 24 (~ 13%) insects, 3 ($\sim$2%) amphibians and terrestrial species, and 4 (~ 2%) fishes and crustaceans. Figure 4 shows four examples of common species from the different groups. The next section explains the utilisation of these data in the assessment of our methodology.

### Evaluation methodology

To evaluate our methodology, a common challenge in Ecological Niche Modelling was simulated, that is, the *prior* assessment of a species’ prevalence in an area before the observation of the species in that area^15,164–168^. Within this challenge, the goal is to assess the presence of the species in an area where surveys have not yet been conducted. This is crucial for models tasked with estimating the *potential* ecological niche of a species, i.e., the species’ habitat within an area different from its native area. In this context, estimating reliable prior information regarding species presence is paramount^26,63,169–172^, because the environmental variables in the native habitat may not fully represent the conditions of the target area due to slight differences in the variables’ ranges^173–176^. Therefore, the results derived from these ENMs should be interpreted with caution and are strongly dependent upon prior probabilities^173,177–180^. If a species is anticipated to be rare in the new habitat, the multiplicative prevalence probability will lead the model to generate more precautionary (stricter) assessments. Conversely, if the species is expected to be common, the model will be inclined to assess a greater suitability.

To simulate this scenario for the 161 selected species of the Massaciuccoli basin, observations from the Massaciuccoli area itself were excluded from the data collection. Instead, observations from analogous habitats were utilised. Specifically, observations from Italian wetlands, as defined by the Ramsar Convention on Wetlands^181^, were collected. These wetlands, represented as a geospatial vector feature file (Fig. 2-right panel), encompassed 57 polygonal areas that were utilised as *datasets* in the feature definitions (section “Feature extraction”). GBIF included a total of 6027 observations in these areas for the 161 species analysed. The threshold for the minimum observations in the *HF* feature was set to 5, corresponding to the 25th percentile of the distribution of the number of species records across the Ramsar wetlands. The time unit for *F* and *HF* was one year. The spatial resolution for *E* was 0.01° (~ 1 *km*).

The target was to classify species as “very common”, “fairly common”, or “rare” through the four models explained in “Classification” (Multi K-means, X-means, VAE, and ensemble assessment). Additionally, a binary assessment was conducted to distinguish between “very common” and “less common” species, merging the “rare” and “fairly common” categories. These assessments were compared against the evaluations made by the two experts on the same categories, who classified the species based on their expertise regarding the basin’s biodiversity. The experts were permitted to consult relevant literature as well as the GBIF occurrence distributions within the Massaciuccoli basin, but they did not have access to the automatic assessments. Furthermore, an additional expert assessment was simulated via a consensus model built upon the evaluations of the two experts. This “expert ensemble” was constructed using the same consensus modelling approach as the “ensemble assessment” method (section “Classification”).

For evaluation metrics, *accuracy* was calculated as the fraction of categories jointly assessed by one method in comparison to each expert’s assessment. In addition, the *agreement* relative to chance was quantified through Cohen’s Kappa^182^.

As an additional evaluation, a *sensitivity analysis* was performed using a *leave-one-out* approach, wherein one feature at a time was omitted from the modelling, and the agreement calculation between the “ensemble assessment” and the “expert ensemble” was repeated. This process ultimately resulted in a ranking of the features based on agreement loss, thereby ordering the features according to their contribution to classification performance.

Finally, the species that were misclassified by all methods were analysed to investigate potential causes of errors.

Overall, the Massaciuccoli basin was used as a representative and ecologically meaningful case study to demonstrate the behaviour and interpretability of the proposed framework. The specific prevalence distributions and class boundaries observed in this system were therefore not intended to be universally applicable, but rather to illustrate how the method operates when applied to a real-world, heterogeneous species assemblage.

### Design choices and alternatives

The proposed methodology relies on several explicit modelling assumptions that warrant discussion. First, it assumes that species prevalence can be meaningfully inferred from a small set of aggregated, ecologically motivated features derived from occurrence records, rather than from species-specific environmental response models. Second, the use of clustering and anomaly-detection techniques assumes that common species share dominant patterns in this feature space, while rare or undersampled species manifest as deviations from these patterns. These assumptions motivate, but do not uniquely determine, the chosen modelling components.

Alternative design choices are possible. For example, different clustering algorithms, distance metrics, or latent representations could be employed, and prevalence thresholds could be defined using various quantile schemes or continuous scoring methods^22^. We expect such alternatives to affect more the exact boundaries between prevalence classes than the overall ordering of species along the common–rare gradient, provided that the feature space remains sufficiently informative and the species set is large enough. The methodology presented here, therefore, represents one principled and reproducible instance within a broader class of unsupervised prevalence assessment approaches, rather than a uniquely determined solution.

## Data Availability

All input and output data, and the software are available on GitHub at https://github.com/cybprojects65/AutomaticSpeciesPrevalenceAssessment . The list of species used in the case study is available within the same repository, at https://github.com/cybprojects65/AutomaticSpeciesPrevalenceAssessment/blob/main/Input/Species_and_groups.csv

## References

[1] Pearson, R. G. Species’ distribution modeling for conservation educators and practitioners. *Synth. Am. Museum Nat. Hist.* **50**, 54–89 (2007).

[2] Jones, M. C., Dye, S. R., Pinnegar, J. K., Warren, R. & Cheung, W. W. Modelling commercial fish distributions: Prediction and assessment using different approaches. *Ecol. Model.* **225**, 133–145 (2012).

[3] Coro, G., Magliozzi, C., Ellenbroek, A., Kaschner, K. & Pagano, P. Automatic classification of climate change effects on marine species distributions in 2050 using the aquamaps model. *Environ. Ecol. Stat.* **23**, 155–180 (2016).

[4] Weber, M. M., Stevens, R. D., Diniz-Filho, J. A. F. & Grelle, C. E. V. Is there a correlation between abundance and environmental suitability derived from ecological niche modelling? a meta-analysis. *Ecography* **40**, 817–828 (2017).

[5] Kosicki, J. Z. Generalised additive models and random forest approach as effective methods for predictive species density and functional species richness. *Environ Ecol. Stat.* **27**, 273–292 (2020).

[6] Huang, S., Zhou, Q., Gu, L., Wang, H. & Zhang, G. Modeling and simulation study of the stoichiometric niche space and niche overlap based on the copula method. *Environ. Ecol. Stat.* **27**, 115–149 (2020).

[7] Deneu, B. et al. Convolutional neural networks improve species distribution modelling by capturing the spatial structure of the environment. *PLOS Comput. Biol.* **17**, e1008856 (2021). 33872302 10.1371/journal.pcbi.1008856 PMC8084334

[8] Hirzel, A. H. & Le Lay, G. Habitat suitability modelling and niche theory. *J. Appl. Ecol.* **45**, 1372–1381 (2008).

[9] Elith, J. & Leathwick, J. R. Species distribution models: Ecological explanation and prediction across space and time. *Ann. Rev. Ecol. Evol. Syst.* **40**, 677–697. 10.1146/annurev.ecolsys.110308.120159 (2009).

[10] Damgaard, C. Modelling ecological presence-absence data along an environmental gradient: Threshold levels of the environment. *Environ. Ecol. Stat.* **13**, 229–236 (2006).

[11] Real, R., Barbosa, A. M. & Vargas, J. M. Obtaining environmental favourability functions from logistic regression. *Environ. Ecol. Stat.* **13**, 237–245 (2006).

[12] Mouton, A. M., De Baets, B. & Goethals, P. L. Ecological relevance of performance criteria for species distribution models. *Ecol. Model.* **221**, 1995–2002 (2010).

[13] Rojas-Soto, O. et al. Calibration areas in ecological niche and species distribution modelling: Unravelling approaches and concepts. *J. Biogeogr.* **51**, 1416–1428 (2024).

[14] Manel, S., Williams, H. C. & Ormerod, S. J. Evaluating presence-absence models in ecology: The need to account for prevalence. *J. Appl. Ecol.* **38**, 921–931 (2001).

[15] Fukuda, S. & De Baets, B. Data prevalence matters when assessing species’ responses using data-driven species distribution models. *Ecol. Inform.* **32**, 69–78 (2016).

[16] Santika, T. Assessing the effect of prevalence on the predictive performance of species distribution models using simulated data. *Global Ecol. Biogeogr.* **20**, 181–192 (2011).

[17] Tarkesh, M. & Jetschke, G. Comparison of six correlative models in predictive vegetation mapping on a local scale. *Environ. Ecol. Stat.* **19**, 437–457 (2012).

[18] Sor, R., Park, Y.-S., Boets, P., Goethals, P. L. & Lek, S. Effects of species prevalence on the performance of predictive models. *Ecol. Model.* **354**, 11–19 (2017).

[19] Phillips, S. J., Anderson, R. P. & Schapire, R. E. Maximum entropy modeling of species geographic distributions. *Ecol. Model.* **190**, 231–259 (2006).

[20] Phillips, S. J. & Dudik, M. Modeling of species distributions with Maxent: New extensions and a comprehensive evaluation. *Ecography* **31**, 161–175 (2008).

[21] Elith, J. et al. A statistical explanation of maxent for ecologists. *Diver. Distrib.* **17**, 43–57 (2011).

[22] Coro, G. et al. Classifying degrees of species commonness: North sea fish as a case study. *Ecol. Model.* **312**, 272–280 (2015).

[23] Lennon, J. J., Beale, C. M., Reid, C. L., Kent, M. & Pakeman, R. J. Are richness patterns of common and rare species equally well explained by environmental variables?. *Ecography* **34**, 529–539. 10.1111/j.1600-0587.2010.06669.x (2011).

[24] Jeliazkov, A. et al. Sampling and modelling rare species: Conceptual guidelines for the neglected majority. *Global Change Biol.* **28**, 3754–3777 (2022). 10.1111/gcb.16114 35098624

[25] Freeman, E. A. & Moisen, G. G. A comparison of the performance of threshold criteria for binary classification in terms of predicted prevalence and kappa. *Ecol. Model.* **217**, 48–58 (2008).

[26] Lawson, C. R., Hodgson, J. A., Wilson, R. J. & Richards, S. A. Prevalence, thresholds and the performance of presence-absence models. *Methods Ecol. Evol.* **5**, 54–64 (2014).

[27] Allouche, O., Tsoar, A. & Kadmon, R. Assessing the accuracy of species distribution models: Prevalence, kappa and the true skill statistic (TSS). *J. Appl. Ecol.* **43**, 1223–1232 (2006).

[28] Wunderlich, R. F., Lin, Y.-P., Anthony, J. & Petway, J. R. Two alternative evaluation metrics to replace the true skill statistic in the assessment of species distribution models. *Nat. Conserv.* **35**, 97–116 (2019).

[29] Grimmett, L., Whitsed, R. & Horta, A. Presence-only species distribution models are sensitive to sample prevalence: Evaluating models using spatial prediction stability and accuracy metrics. *Ecol. Model.* **431**, 109194 (2020).

[30] McPherson, J., Jetz, W. & Rogers, D. J. The effects of species’ range sizes on the accuracy of distribution models: Ecological phenomenon or statistical artefact?. *J. Appl. Ecol.* **41**, 811–823 (2004).

[31] Enquist, B. J. et al. The commonness of rarity: Global and future distribution of rarity across land plants. *Sci. Adv.* **5**, eaaz0414. 10.1126/sciadv.aaz0414 (2019). 31807712 10.1126/sciadv.aaz0414 PMC6881168

[32] Sillero, N. et al. Want to model a species niche? a step-by-step guideline on correlative ecological niche modelling. *Ecol. Model.* **456**, 109671 (2021).

[33] Balbuena, J. A. et al. Fuzzy quantification of common and rare species in ecological communities (fuzzyq). *Methods Ecol. Evol.* **12**, 1070–1079. 10.1111/2041-210X.13588 (2021).

[34] Lane, M. A. & Edwards, J. L. The global biodiversity information facility (GBIF). *Syst. Assoc. Spec.* **73**, 1 (2007) (**gbif.org**).

[35] Ciccolini, V. et al. Restoration of a mediterranean drained peatland: the case study of the massaciuccoli lake basin (tuscany, it). In *Extended abstract) in International Workshop AWARE Approaches in Wetland Restoration*, 21–23 (2013).

[36] Nikologianni, A. Exploring the future: Landscape architects and emerging professionals. *Common Ground* **1**, 47–47 (2019).

[37] Bertacchi, A. et al. A case of ecological renaturation in a drained mediterranean peatland: the case study of the massaciuccoli lake basin (tuscany, it). In *Congresso della Societ*à *Botanica Italiana onlus Pavia, 14-17 September 2015: ABSTRACTS KEYNOTE LECTURES, COMMUNICATIONS, POSTERS*, 16–16 (Società Botanica Italiana, 2015).

[38] Giugliano, L. et al. The dragonflies of the retrodunal wetlands in the migliarino, san rossore, massaciuccoli regional park (odonata). *Bollettino della Società Entomologica Italiana* **143**, 3–13 (2011).

[39] Colombini, I., Cimò, F. & Chelazzi, L. *Assessment of local biodiversity Macroinvertebrate diversity*, chap. 3, 59–78 (Demetra, Florence, Italy, 2013).

[40] Colombaroli, D. & Tinner, W. Determining the long-term changes in biodiversity and provisioning services along a transect from central europe to the mediterranean. *The Holocene* **23**, 1625–1634 (2013).

[41] Finlayson, C. M., Davidson, N., Pritchard, D., Milton, G. R. & MacKay, H. The Ramsar convention and ecosystem-based approaches to the wise use and sustainable development of wetlands. *J. Int. Wildlife Law Policy* **14**, 176–198. 10.1080/13880292.2011.626704 (2011).

[42] Gaston, K. J. et al. Abundance-occupancy relationships. *J. Appl. Ecol.* **37**, 39–59 (2000).

[43] Flather, C. H. & Sieg, C. H. Species rarity: Definition, causes and classification. Conservation of rare or little-known species: Biological, social, and economic considerations 40–66 (2007).

[44] Matthews, T. J. & Whittaker, R. J. On the species abundance distribution in applied ecology and biodiversity management. *J. Appl. Ecol.* **52**, 443–454 (2015).

[45] Magurran, A. E. Biodiversity in the context of ecosystem function. *Marine Biodiv. Ecosyst. Funct. Frameworks, Methodol. Integration* **16**, 23 (2012).

[46] Ricotta, C. et al. From abundance-based to functional-based indicator species. *Ecol. Indicators* **118**, 106761 (2020).

[47] Yan, P. et al. The essential role of biodiversity in the key axes of ecosystem function. *Global Change Biol.* **29**, 4569–4585 (2023). 10.1111/gcb.16666 36880889

[48] Mi, X. et al. The contribution of rare species to community phylogenetic diversity across a global network of forest plots. *Am. Nat.* **180**, E17–E30 (2012). 22673660 10.1086/665999

[49] Mouillot, D. et al. Rare species support vulnerable functions in high-diversity ecosystems. *PLoS Biol.* **11**, e1001569 (2013). 23723735 10.1371/journal.pbio.1001569 PMC3665844

[50] Coro, G., Pagano, P. & Ellenbroek, A. Combining simulated expert knowledge with neural networks to produce ecological niche models for latimeria chalumnae. *Ecol. Model.* **268**, 55–63 (2013).

[51] Coro, G., Magliozzi, C., Ellenbroek, A. & Pagano, P. Improving data quality to build a robust distribution model for architeuthis dux. *Ecol. Model.* **305**, 29–39 (2015).

[52] Bagousse-Pinguet, Y. L. et al. Functional rarity and evenness are key facets of biodiversity to boost multifunctionality. *Proc. Nat. Acad. Sci.* **118**, e2019355118. 10.1073/pnas.2019355118 (2021). 33568533 10.1073/pnas.2019355118 PMC7896339

[53] Gaston, K. J. & Fuller, R. A. Commonness, population depletion and conservation biology. *Trends Ecol. Evol.* **23**, 14–19 (2008). 18037531 10.1016/j.tree.2007.11.001

[54] Gaston, K. J. Valuing common species. *Science* **327**, 154–155 (2010). 20056880 10.1126/science.1182818

[55] Gaston, K. J. Common ecology. *BioScience* **61**, 354–362 (2011).

[56] Brasil, L. S. et al. The importance of common and the irrelevance of rare species for partition the variation of community matrix: Implications for sampling and conservation. *Sci. Rep.* **10**, 19777 (2020). 33188230 10.1038/s41598-020-76833-5 PMC7666184

[57] Gonzalez, A. et al. Scaling-up biodiversity-ecosystem functioning research. *Ecol. Lett.* **23**, 757–776 (2020). 31997566 10.1111/ele.13456 PMC7497049

[58] Eger, A. M., Best, R. J. & Baum, J. K. Dominance determines fish community biomass in a temperate seagrass ecosystem. *Ecol. Evol.* **11**, 10489–10501 (2021). 34367591 10.1002/ece3.7854 PMC8328455

[59] Lisner, A., Konečná, M., Blažek, P. & Lepš, J. Community biomass is driven by dominants and their characteristics-the insight from a field biodiversity experiment with realistic species loss scenario. *J. Ecol.* **111**, 240–250 (2023).

[60] Coro, G. et al. Climate change effects on animal presence in the massaciuccoli lake basin. *Ecol. Inform.* **81**, 102644. 10.1016/j.ecoinf.2024.102644 (2024).

[61] Takolander, A. et al. Cross-realm transferability of species distribution models-species characteristics and prevalence matter more than modelling methods applied. *Ecol. Model.* **499**, 110950 (2025).

[62] Phillips, S. J. & Dudík, M. Modeling of species distributions with maxent: new extensions and a comprehensive evaluation. *Ecography* **31**, 161–175 (2008).

[63] Vermeiren, P., Reichert, P. & Schuwirth, N. Integrating uncertain prior knowledge regarding ecological preferences into multi-species distribution models: Effects of model complexity on predictive performance. *Ecol. Model.* **420**, 108956. 10.1016/j.ecolmodel.2020.108956 (2020).

[64] Duarte, A., Spaan, R. S., Peterson, J. T., Pearl, C. A. & Adams, M. J. Bayesian networks facilitate updating of species distribution and habitat suitability models. *Ecol. Model* **501**, 110982. 10.1016/j.ecolmodel.2024.110982 (2025).

[65] Saffer, A. et al. Quantifying uncertainty in forecasts of when and where invasions happen. *Biol. Invasions* **27**, 117 (2025).

[66] Chapin Iii, F. S. et al. Consequences of changing biodiversity. *Nature* **405**, 234–242 (2000). 10821284 10.1038/35012241

[67] Dostál, P., Tasevová, K. & Klinerová, T. Linking species abundance and overyielding from experimental communities with niche and fitness characteristics. *J. Ecol.* **107**, 178–189 (2019).

[68] Engel, T. et al. How does variation in total and relative abundance contribute to gradients of species diversity?. *Ecol. Evol.* **12**, e9196 (2022). 35991281 10.1002/ece3.9196 PMC9382643

[69] Kerr, J. T., Kharouba, H. M. & Currie, D. J. The macroecological contribution to global change solutions. *Science* **316**, 1581–1584 (2007). 17569854 10.1126/science.1133267

[70] Núñez-Antonio, G., Mendoza, M., Contreras-Cristán, A., Gutiérrez-Peña, E. & Mendoza, E. Bayesian nonparametric inference for the overlap of daily animal activity patterns. *Environ. Ecol. Stat.* **25**, 471–494 (2018).

[71] Sparrow, B. D. et al. Effective ecosystem monitoring requires a multi-scaled approach. *Biol. Rev.* **95**, 1706–1719. 10.1111/brv.12636 (2020). 32648358 10.1111/brv.12636 PMC7689690

[72] Ray, A. M., Murphy, M. A. & Hossack, B. R. Long-term monitoring of a species suite of ecological indicators: a coordinated conservation framework for the greater yellowstone ecosystem. *Ecol. Indicat.* **137**, 108774 (2022).

[73] Dornelas, M. et al. Assemblage time series reveal biodiversity change but not systematic loss. *Science* **344**, 296–299 (2014). 24744374 10.1126/science.1248484

[74] Coro, G., Sana, L. & Bove, P. An open science automatic workflow for multi-model species distribution estimation. *Int. J. Data Sci. Anal.* **15**, 89 (2024).

[75] Prima, M.-C. et al. A comprehensive framework to assess multi-species landscape connectivity. *Methods Ecol. Evol.* **15**, 2385–2399 (2024).

[76] Harding, P. The national biodiversity network in the UK. Accessed on Sept. 2025 at https://repository.naturalis.nl/pub/219861/ (2025).

[77] Grassle, J. F. The ocean biogeographic information system (OBIS): An on-line, worldwide atlas for accessing, modeling and mapping marine biological data in a multidimensional geographic context. *Oceanography* **13**, 5–7 (2000).

[78] Isaac, N. J., van Strien, A. J., August, T. A., de Zeeuw, M. P. & Roy, D. B. Statistics for citizen science: Extracting signals of change from noisy ecological data. *Methods Ecol. Evol.* **5**, 1052–1060 (2014).

[79] Maldonado, C. et al. Estimating species diversity and distribution in the era of big data: To what extent can we trust public databases?. *Global Ecol. Biogeogr* **24**, 973–984 (2015). 10.1111/geb.12326 PMC5012125 27656106

[80] Roll, U. et al. The global distribution of tetrapods reveals a need for targeted reptile conservation. *Nat. Ecol. Evol.* **1**, 1677–1682 (2017). 28993667 10.1038/s41559-017-0332-2

[81] Heberling, J. M., Miller, J. T., Noesgaard, D., Weingart, S. B. & Schigel, D. Data integration enables global biodiversity synthesis. *Proc. Nat. Acad. Sci.* **118**, e2018093118 (2021). 33526679 10.1073/pnas.2018093118 PMC8017944

[82] Tang, B., Clark, J. S. & Gelfand, A. E. Modeling spatially biased citizen science effort through the Ebird database. *Environ. Ecol. Stat.* **28**, 609–630 (2021).

[83] Baker, D. J., Maclean, I. M., Goodall, M. & Gaston, K. J. Species distribution modelling is needed to support ecological impact assessments. *J. Appl. Ecol.* **58**, 21–26 (2021).

[84] Tehrani, N. A., Naimi, B. & Jaboyedoff, M. A data-integration approach to correct sampling bias in species distribution models using multiple datasets of breeding birds in the swiss alps. *Ecol. Inform.* **69**, 101501. 10.1016/j.ecoinf.2021.101501 (2022).

[85] Rathore, M. K. & Sharma, L. K. Efficacy of species distribution models (SDMS) for ecological realms to ascertain biological conservation and practices. *Biodiver. Conserv.* **32**, 3053–3087 (2023).

[86] Rios, E. B., Sadler, J., Graham, L. & Matthews, T. J. Species distribution models and island biogeography: Challenges and prospects. *Global Ecol. Conserv.* **51**, e02943 (2024).

[87] Benavides Rios, E., Sadler, J., Graham, L. & Matthews, T. J. Species distribution models and island biogeography: Challenges and prospects. *Global Ecol. Conserv.* **51**, e02943. 10.1016/j.gecco.2024.e02943 (2024).

[88] Barbosa, W. L. & Alves-Souza, S. N. Data quality issues in data used in species distribution models: A systematic literature review. *Ecol. Inform.* **91**, 103378. 10.1016/j.ecoinf.2025.103378 (2025).

[89] Fung, T., Villain, L. & Chisholm, R. A. Analytical formulae for computing dominance from species-abundance distributions. *J. Theor. Biol.* **386**, 147–158. 10.1016/j.jtbi.2015.09.011 (2015). 26409166 10.1016/j.jtbi.2015.09.011

[90] Liu, M. & Samal, A. A fuzzy clustering approach to delineate agroecozones. *Ecol. Model.* **149**, 215–228 (2002).

[91] Dale, M. B., Dale, P. & Tan, P. Supervised clustering using decision trees and decision graphs: An ecological comparison. *Ecol. Model.* **204**, 70–78 (2007).

[92] Debeljak, M. et al. Analysis of time series data on agroecosystem vegetation using predictive clustering trees. *Ecol. Model.* **222**, 2524–2529 (2011).

[93] Picard, N. & Bar-Hen, A. Cluster analysis for two-level data sets: Classifying tree species from individual measurements. *Ecol. Inform.* **15**, 1–7. 10.1016/j.ecoinf.2013.02.001 (2013).

[94] Pang, S. E. H., Slik, J. W. F., Zurell, D. & Webb, E. L. The clustering of spatially associated species unravels patterns in tropical tree species distributions. *Ecosphere* **14**, e4589. 10.1002/ecs2.4589 (2023).

[95] Hefley, T. J., Tyre, A. J., Baasch, D. M. & Blankenship, E. E. Nondetection sampling bias in marked presence-only data. *Ecol. Evol.* **3**, 5225–5236 (2013). 24455151 10.1002/ece3.887 PMC3892331

[96] Fithian, W., Elith, J., Hastie, T. & Keith, D. A. Bias correction in species distribution models: Pooling survey and collection data for multiple species. *Methods Ecol. Evol.* **6**, 424–438 (2015). 27840673 10.1111/2041-210X.12242 PMC5102514

[97] Twining, J. P. et al. Integrating presence-only and detection/non-detection data to estimate distributions and expected abundance of difficult-to-monitor species on a landscape-scale. *J. Appl. Ecol.* **61**, 1441–1459 (2024).

[98] Beale, C. M. & Lennon, J. J. Incorporating uncertainty in predictive species distribution modelling. *Philos. Trans. R. Soc. B: Biol. Sci.* **367**, 247–258 (2012). 10.1098/rstb.2011.0178 PMC3223803 22144387

[99] Hanberry, B. & He, H. Prevalence, statistical thresholds, and accuracy assessment for species distribution models. *Web Ecol.* **13**, 13–19 (2013).

[100] Rubbens, P. et al. Machine learning in marine ecology: An overview of techniques and applications. *ICES J. Marine Sci.* **80**, 1829–1853 (2023).

[101] Fleiss, J. L. Measuring nominal scale agreement among many raters. *Psychol. Bull.* **76**, 378 (1971).

[102] Brichetti, P., Grattini, N. & Lui, F. Distribuzione e consistenza delle popolazioni nidificanti di forapaglie comune Acrocephalus schoenobaenus in Italia. *AVOCETTA* **29**, 19 (2005).

[103] Fishbase. Cyprinus carpio (Linnaeus, 1758) (2025).

[104] Alessio, G., Duchi, A., Bercelli, M., Baldacchini, G. & Bianuccì, P. Interrelazione tra ittiofauna ed eutrofizzazione r nel lago di massaciuccoli (toscana) (2019).

[105] Jacobs, S. Brown marmorated stink bug (2015).

[106] Cianferoni, F., Graziani, F., Dioli, P. & Ceccolini, F. Review of the occurrence of halyomorpha halys (hemiptera: Heteroptera: Pentatomidae) in Italy, with an update of its european and world distribution. *Biologia* **73**, 599–607 (2018).

[107] Corti, C. et al. Species diversity and distribution of amphibians and reptiles in Sardinia, italy. Acta Herpetologica **17** (2022).

[108] Luan, J. et al. Matching data types to the objectives of species distribution modeling: An evaluation with marine fish species. *Front. Marine Sci.* **8**, 771071 (2021).

[109] Ahmed, N., Atzberger, C. & Zewdie, W. Species distribution modelling performance and its implication for sentinel-2-based prediction of invasive prosopis juliflora in lower awash river basin, ethiopia. *Ecol. Process.* **10**, 18 (2021).

[110] Wilkinson, D. P., Golding, N., Guillera-Arroita, G., Tingley, R. & McCarthy, M. A comparison of predictive performance of joint species distribution models for presence-absence data. EcoEvoRxiv 1–31 (2023).

[111] Tytar, V. et al. Species distribution modeling of ixodes ricinus (linnaeus, 1758) under current and future climates, with a special focus on latvia and ukraine. *Climate* **12**, 184 (2024).

[112] Anselmetto, N. et al. Species distribution models built with local species data perform better for current time, but suffer from niche truncation. *Agric. Forest Meteorol.* **362**, 110361 (2025).

[113] Coro, G. Open science and artificial intelligence supporting blue growth. Environ. Eng. Manag. J. (EEMJ) **19** (2020).

[114] Queiroz, N. et al. Reply to: Caution over the use of ecological big data for conservation. *Nature* **595**, E20–E28 (2021). 34234328 10.1038/s41586-021-03464-9

[115] Zennaro, F. et al. Exploring machine learning potential for climate change risk assessment. *Earth-Sci. Rev.* **220**, 103752 (2021).

[116] Coro, G. An open science oriented Bayesian interpolation model for marine parameter observations. *Environ. Model. Softw.* **172**, 105901 (2024).

[117] Pavirani, L., Bove, P. & Coro, G. Assessing marine ecosystem risks through unsupervised methods. *Ecol. Inform.* **90**, 103334. 10.1016/j.ecoinf.2025.103334 (2025).

[118] Trakhtenbrot, A. & Kadmon, R. Effectiveness of environmental cluster analysis in representing regional species diversity. *Conserv. Biol.* **20**, 1087–1098. 10.1111/j.1523-1739.2006.00500.x (2006). 16922225 10.1111/j.1523-1739.2006.00500.x

[119] Tichý, L., Chytrý, M., Hájek, M., Talbot, S. S. & Botta-Dukát, Z. Optimclass: Using species-to-cluster fidelity to determine the optimal partition in classification of ecological communities. *Journal of Vegetation Science* **21**, 287–299. 10.1111/j.1654-1103.2009.01143.x (2010).

[120] Maturo, F. Unsupervised classification of ecological communities ranked according to their biodiversity patterns via a functional principal component decomposition of hill’s numbers integral functions. *Ecol. Indicat.* **90**, 305–315. 10.1016/j.ecolind.2018.03.013 (2018).

[121] Luo, M. et al. The use of global biodiversity information facility (GBIF)-mediated data in publications written in Chinese. *Global Ecol. Conserv.* **25**, e01406. 10.1016/j.gecco.2020.e01406 (2021).

[122] Ivanova, N. & Shashkov, M. The possibilities of GBIF data use in ecological research. *Rus. J. Ecol.* **52**, 1–8 (2021).

[123] Wieczorek, J. et al. Darwin core: An evolving community-developed biodiversity data standard. *PloS One* **7**, e29715 (2012). 22238640 10.1371/journal.pone.0029715 PMC3253084

[124] Candela, L. et al. An infrastructure-oriented approach for supporting biodiversity research. *Ecol. Inform.* **26**, 162–172 (2015).

[125] Coro, G., Panichi, G., Scarponi, P. & Pagano, P. Cloud computing in a distributed e-infrastructure using the web processing service standard. *Concurr. Comput. Pract. Exp* **29**, e4219 (2017).

[126] Assante, M. et al. Enacting open science by d4science. *Fut. Gener. Comput. Syst.* **101**, 555–563 (2019).

[127] Assante, M. et al. Virtual research environments co-creation: The d4science experience. Concurrency and Computation: Practice and Experience e6925 (2022).

[128] Nugent, J. iNaturalist: Citizen science for 21st-century naturalists. *Sci. Scope* **41**, 12–15 (2018).

[129] Sullivan, B. L. et al. The ebird enterprise: An integrated approach to development and application of citizen science. *Biol. Conserv.* **169**, 31–40 (2014).

[130] rgbif. An interface to the GBIF API for the R statistical programming environment. Available at https://www.gbif.org/tool/81747/rgbif (2025).

[131] Blackburn, T. M., Cassey, P. & Gaston, K. J. Variations on a theme: Sources of heterogeneity in the form of the interspecific relationship between abundance and distribution. *J. Animal Ecol.* **75**, 1426–1439 (2006). 10.1111/j.1365-2656.2006.01167.x 17032375

[132] Pearman, P. B. & Weber, D. Common species determine richness patterns in biodiversity indicator taxa. *Biol. Conserv.* **138**, 109–119 (2007).

[133] McGill, B. J. et al. Species abundance distributions: moving beyond single prediction theories to integration within an ecological framework. *Ecol. Lett.* **10**, 995–1015 (2007). 17845298 10.1111/j.1461-0248.2007.01094.x

[134] Gaston, K. J. Valuing Common Species. *Science* **327**, 154–155. 10.1126/science.1182818 (2010). 20056880 10.1126/science.1182818

[135] Webb, T. J., Freckleton, R. P. & Gaston, K. J. Characterizing abundance-occupancy relationships: There is no artefact. *Global Ecol. Biogeogr.* **21**, 952–957 (2012).

[136] Webb, T. J. Marine and terrestrial ecology: unifying concepts, revealing differences. *Trends Ecol. Evol.* **27**, 535–541 (2012). 22795608 10.1016/j.tree.2012.06.002

[137] Connolly, S. R. et al. Commonness and rarity in the marine biosphere. Proceedings of the National Academy of Sciences 201406664 (2014). 10.1073/pnas.1406664111 PMC4060690 24912168

[138] Hughes, T. P., Bellwood, D. R., Connolly, S. R., Cornell, H. V. & Karlson, R. H. Double jeopardy and global extinction risk in corals and reef fishes. *Curr. Biol.* **24**, 2946–2951. 10.1016/j.cub.2014.10.037 (2014). 25454782 10.1016/j.cub.2014.10.037

[139] Maturo, F. Unsupervised classification of ecological communities ranked according to their biodiversity patterns via a functional principal component decomposition of hill’s numbers integral functions. *Ecol. Indicators* **90**, 305–315 (2018).

[140] MacLeod, C. D. Habitat representativeness score (hrs): A novel concept for objectively assessing the suitability of survey coverage for modelling the distribution of marine species. *J. Marine Biol. Assoc. UK* **90**, 1269–1277 (2010).

[141] Guala, G. Integrated taxonomic information system (itis). Available at https://itis.gov/ (2019).

[142] Magliozzi, C., Coro, G., Grabowski, R. C., Packman, A. I. & Krause, S. A multiscale statistical method to identify potential areas of hyporheic exchange for river restoration planning. *Environ. Model. Softw.* **111**, 311–323 (2019).

[143] Coro, G., Pavirani, L. & Ellenbroek, A. Computing ecosystem risk hotspots: A mediterranean case study. *Ecol. Inform.* **85**, 102918 (2025).

[144] Pavirani, L., Bove, P. & Coro, G. Ecosystem risk assessment through stressor concurrency identification: A comparative analysis. In *OCEANS 2025 Brest*, 1–6. 10.1109/OCEANS58557.2025.11104682 (2025).

[145] Schwarz, G. et al. Estimating the dimension of a model. *Ann. Stat.* **6**, 461–464 (1978).

[146] Neath, A. A. & Cavanaugh, J. E. The bayesian information criterion: background, derivation, and applications. *Wiley Interdisciplinary Rev. Comput. Stat.* **4**, 199–203 (2012).

[147] Pelleg, D. & Moore, A. W. X-means: Extending k-means with efficient estimation of the number of clusters. In *Proceedings of the Seventeenth International Conference on Machine Learning*, ICML ’00, 727–734 (Morgan Kaufmann Publishers Inc., San Francisco, CA, USA, 2000).

[148] Kingma, D. P. & Welling, M. *Auto-encoding variational bayes* **1312**, 6114 (2014).

[149] Zhang, C. & Peng, Y. Stacking vae and gan for context-aware text-to-image generation. In *2018 IEEE Fourth International Conference on Multimedia Big Data (BigMM)*, 1–5. IEEE (IEEE, Xi’an, China, 2018).

[150] Lin, S. et al. Anomaly detection for time series using vae-lstm hybrid model. In *ICASSP 2020 - 2020 IEEE International Conference on Acoustics, Speech and Signal Processing (ICASSP)*, 4322–4326, 10.1109/ICASSP40776.2020.9053558 (IEEE, Barcelona, Spain, 2020).

[151] Liu, K. et al. Cells image generation method based on vae-sgan. *Proc. Comput. Sci.* **183**, 589–595 (2021).

[152] Mahmud, M. S., Huang, J. Z. & Fu, X. Variational autoencoder-based dimensionality reduction for high-dimensional small-sample data classification. *Int. J. Comput. Intel. Appl.* **19**, 2050002 (2020).

[153] Rundo, L. & Militello, C. Image biomarkers and explainable AI: Handcrafted features versus deep learned features. *Eur. Radiol. Exp.* **8**, 130 (2024). 39560820 10.1186/s41747-024-00529-y PMC11576747

[154] Baneschi, I. *Geochemical and environmental study of a coastal ecosystem: Massaciuccoli lake (northern Tuscany, Italy)*. Ph.D. thesis, UNIVERSITÀ CA’FOSCARI VENEZIA; Università degli studi Ca’Foscari di Venezia (2007).

[155] Rossetto, R. et al. Surface water and groundwater monitoring and numerical modeling of the southern sector of the massaciuccoli lake basin (italy). *Rendiconti Online Società Geologica Italiana* **11**, 189–190 (2010).

[156] Rossetto, R. et al. Water management sustainability in reclaimed coastal areas. the case of the massaciuccoli lake basin (tuscany, italy). In *EGU General Assembly Conference Abstracts*, 12158 (2010).

[157] Silvestri, N. et al. 6.1. study case 1: Restoration of an agricultural drained peatland: the case study of the massaciuccoli lake basin in tuscany (italy). In *Best Practices in Evaluation and Restoration of Degraded Mediterranean Environments*, 95–101 (IBADER, Santiago de Compostela, Spain, 2019).

[158] Pignalosa, A. et al. Long-term simulations of nature-based solutions effects on runoff and soil losses in a flat agricultural area within the catchment of lake massaciuccoli (central italy). *Agric. Water Manag.* **273**, 107870 (2022).

[159] Pignalosa, A. et al. Modelling the effects of nbs adoption in mitigating soil losses of a land reclamation area in the massaciuccoli lake catchment (central italy). In *EGU General Assembly Conference Abstracts*, EGU22–7965 (2022).

[160] Regione Toscana. Contratto di Lago per il Massaciuccoli. Available at https://partecipa.toscana.it/web/contratto-di-lago-per-il-massaciuccoli (2018).

[161] Colombaroli, D., Marchetto, A. & Tinner, W. Long-term interactions between mediterranean climate, vegetation and fire regime at lago di massaciuccoli (tuscany, italy). *J. Ecol.* **95**, 755–770 (2007).

[162] Tinner, W. et al. The past ecology of abies alba provides new perspectives on future responses of silver fir forests to global warming. *Ecol. Monogr.* **83**, 419–439. 10.1890/12-2231.1 (2013).

[163] Lastrucci, L. et al. Contribution to the knowledge of the vegetation of the lake massaciuccoli (northern tuscany, italy). *Plant Sociol.* **54**, 67–87 (2017).

[164] Lachish, S., Gopalaswamy, A. M., Knowles, S. C. L. & Sheldon, B. C. Site-occupancy modelling as a novel framework for assessing test sensitivity and estimating wildlife disease prevalence from imperfect diagnostic tests. *Methods Ecol. Evolut.* **3**, 339–348. 10.1111/j.2041-210X.2011.00156.x (2012).

[165] Pagel, J. & Schurr, F. M. Forecasting species ranges by statistical estimation of ecological niches and spatial population dynamics. *Global Ecol. Biogeogr.* **21**, 293–304. 10.1111/j.1466-8238.2011.00663.x (2012).

[166] Agardy, T., Di Sciara, G. N. & Christie, P. Mind the gap: addressing the shortcomings of marine protected areas through large scale marine spatial planning. *Marine Policy* **35**, 226–232 (2011).

[167] Sor, R., Park, Y.-S., Boets, P., Goethals, P. L. & Lek, S. Effects of species prevalence on the performance of predictive models. *Ecol. Model.* **354**, 11–19. 10.1016/j.ecolmodel.2017.03.006 (2017).

[168] Pili, A. N., Tingley, R., Sy, E. Y., Diesmos, M. L. L. & Diesmos, A. C. Niche shifts and environmental non-equilibrium undermine the usefulness of ecological niche models for invasion risk assessments. *Sci. Reports* **10**, 7972 (2020). 10.1038/s41598-020-64568-2 PMC7224218 32409706

[169] Piekielek, N. B., Hansen, A. & Chang, T. Using custom scientific workflow software and GIS to inform protected area climate adaptation planning in the greater yellowstone ecosystem. *Ecol. Inform.* **30**, 40–48 (2015).

[170] Citores, L. et al. Modelling species presence–absence in the ecological niche theory framework using shape-constrained generalized additive models. *Ecol. Model.* **418**, 108926. 10.1016/j.ecolmodel.2019.108926 (2020).

[171] Konowalik, K. & Nosol, A. Evaluation metrics and validation of presence-only species distribution models based on distributional maps with varying coverage. *Sci. Rep.* **11**, 1482 (2021). 33452285 10.1038/s41598-020-80062-1 PMC7811024

[172] Santini, L., Benítez-López, A., Maiorano, L., Čengić, M. & Huijbregts, M. A. Assessing the reliability of species distribution projections in climate change research. *Div. Distrib.* **27**, 1035–1050 (2021).

[173] Elith, J. et al. A statistical explanation of MaxEnt for ecologists. *Div. Distrib.* **17**, 43–57. 10.1111/j.1472-4642.2010.00725.x (2011).

[174] Wan, J.-Z., Wang, C.-J. & Yu, F.-H. Effects of occurrence record number, environmental variable number, and spatial scales on maxent distribution modelling for invasive plants. *Biologia* **74**, 757–766 (2019).

[175] Mushtaq, S., Reshi, Z. A., Shah, M. A. & Charles, B. Modelled distribution of an invasive alien plant species differs at different spatiotemporal scales under changing climate: a case study of parthenium hysterophorus l. *Trop. Ecol.* **62**, 398–417 (2021).

[176] Coro, G., Bove, P. & Ellenbroek, A. Habitat distribution change of commercial species in the Adriatic sea during the covid-19 pandemic. *Ecol. Inform.* **69**, 101675 (2022). 35615467 10.1016/j.ecoinf.2022.101675 PMC9123804

[177] Stohlgren, T. J., Jarnevich, C. S., Esaias, W. E. & Morisette, J. T. Bounding species distribution models. *Curr. Zool.* **57**, 642–647 (2011).

[178] Owens, H. L. et al. Constraints on interpretation of ecological niche models by limited environmental ranges on calibration areas. *Ecol. Model.* **263**, 10–18 (2013).

[179] Kumar, S., Graham, J., West, A. M. & Evangelista, P. H. Using district-level occurrences in maxent for predicting the invasion potential of an exotic insect pest in India. *Comput. Electron. Agric.* **103**, 55–62 (2014).

[180] Holt, C. D. S., Nevin, O. T., Smith, D. & Convery, I. Environmental niche overlap between snow leopard and four prey species in Kazakhstan. *Ecol. Informat.* **48**, 97–103 (2018).

[181] Finlayson, M., Davidson, N., Pritchard, D., Milton, R. & MacKay, H. The Ramsar convention and ecosystem-based approaches to the wise use and sustainable development of wetlands. *J. Int. Wildlife Law Policy* **14**, 176–198 (2011).

[182] Cohen, J. et al. A coefficient of agreement for nominal scales. *Educ. Psychol. Meas.* **20**, 37–46 (1960).

